# Theoretical and Kinetic Tools for Selecting Effective Antioxidants: Application to the Protection of Omega-3 Oils with Natural and Synthetic Phenols

**DOI:** 10.3390/ijms17081220

**Published:** 2016-07-29

**Authors:** Romain Guitard, Véronique Nardello-Rataj, Jean-Marie Aubry

**Affiliations:** Univ. Lille, CNRS, Centrale Lille, ENSCL, Univ. Artois, UMR 8181–UCCS-Unité de Catalyse et Chimie du Solide, F-59000 Lille, France; romain.guitard@live.fr

**Keywords:** natural and synthetic phenols, antioxidant, bond dissociation enthalpy (BDE), 2,2-diphenyl-1-picrylhydrazyl (DPPH^•^), omega-3 fatty acid methyl esters (FAMEs), linseed oil, autoxidation, stoichiometric number

## Abstract

Radical-scavenging antioxidants play crucial roles in the protection of unsaturated oils against autoxidation and, especially, edible oils rich in omega-3 because of their high sensitivity to oxygen. Two complementary tools are employed to select, among a large set of natural and synthetic phenols, the most promising antioxidants. On the one hand, density functional theory (DFT) calculations provide bond dissociation enthalpies (BDEs) of 70 natural (i.e., tocopherols, hydroxybenzoic and cinnamic acids, flavonoids, stilbenes, lignans, and coumarins) and synthetic (i.e., 2,6-di*-tert*-butyl-4-methylphenol (BHT), 3-*tert*-butyl-4-hydroxyanisol (BHA), and *tert*-butylhydroquinone (TBHQ)) phenols. These BDEs are discussed on the basis of structure–activity relationships with regard to their potential antioxidant activities. On the other hand, the kinetic rate constants and number of hydrogen atoms released per phenol molecule are measured by monitoring the reaction of phenols with 2,2-diphenyl-1-picrylhydrazyl (DPPH^•^) radical. The comparison of the results obtained with these two complementary methods allows highlighting the most promising antioxidants. Finally, the antioxidant effectiveness of the best candidates is assessed by following the absorption of oxygen by methyl esters of linseed oil containing 0.5 mmol L^−1^ of antioxidant and warmed at 90 °C under oxygen atmosphere. Under these conditions, some natural phenols namely epigallocatechin gallate, myricetin, rosmarinic and carnosic acids were found to be more effective antioxidants than α-tocopherol.

## 1. Introduction

Omega-3 essential fatty acids have drawn attention of scientists for many years and studies have multiplied in recent decades, highlighting their virtues and mandatory character to the proper functioning of human bodies [1,2]. Nevertheless, due to their large number of unsaturations, omega-3 oils are highly oxidizable. This process plays an important role in the degradation of the organoleptic properties of food. All lipids containing unsaturated fatty acids such as vegetable oils, fish oils, animal fats, cell membranes and lipoproteins are concerned with lipid peroxidation. In recent decades, mechanistic studies of lipid peroxidation have known a renewed interest because of their implication in the field of nutrition.

Unsaturated lipids (LH) are prone to autoxidation, which takes place in three main steps. The first one is the initiation step which consists of the loss of a hydrogen atom triggered by metal traces, light or heat (Equation (1)). The resulting lipid radical (L^•^) reacts with fundamental oxygen (^3^O_2_) in a second step to form a peroxyl radical (LOO^•^) (Equation (2)). During the propagation stage, LOO^•^ reacts with LH to form fatty acid hydroperoxides (LOOH) which are primary oxidation products (Equation (3)). In a third step, i.e., the termination step, two radicals react together to form non-radical products (Equations (4)–(6)) [3,4]. Hydroperoxides are unstable compounds that lead to alcoxyl (LO^•^) and peroxyl (LOO^•^) radicals which further form other oxidized products such as alcohols, aldehydes and ketones. One possible decomposition of lipid hydroperoxides is known as the Russel mechanism in which the combination of two peroxyl radicals LOO^•^ provides a ketone L(O), an alcohol LOH and singlet molecular oxygen ^1^O_2_ which can take place in biological systems (Equation (4)). Cyclisation mechanisms can also be involved in the formation of cyclic peroxides [5].

*Initiation*

(1) $$\text{LH}\overset{\text{Initiator}}{\to }\;L^{•}\;+\;H^{•}$$

*Propagation*

(2) $$L^{•}\;+\;{}^{3}O_{2}\to {\text{LOO}}^{•}$$

(3) $${\text{LOO}}^{•}\;+\text{LH}\to \text{LOOH}+\;L^{•}$$

*Termination*

(4) $${\text{LOO}}^{•}\;+\;{\text{LOO}}^{•}\to \text{L}(\text{O})+\text{LOH}+\;{}^{1}O_{2}$$

(5) $${\text{LOO}}^{•}\;+\;L^{•}\to \text{LOOL}$$

(6) $$L^{•}\;+\;L^{•}\to \text{LL}$$

In order to reduce the damages of these free radicals on food and biological systems, scientists are searching effective and non-toxic antioxidants [6]. Different factors influence the antioxidant power of phenols [7]: (i) Low value of Bond Dissociation Enthalpy (BDE) of the phenolic bond favors the transfer of the phenolic hydrogen to free radicals (i.e., R^•^, RO^•^ and ROO^•^) [8,9,10,11,12,13]; (ii) High value of ionization potential (IP) avoids the transfer of electron from phenols to oxygen. Consequently, the pro-oxidant potential of the antioxidant is reduced [7,11,14,15,16,17]; (iii) high solubility of the phenol into the protected medium improves the antioxidant power [18,19].

There are numerous experimental and theoretical investigations dealing with bond dissociation enthalpies (BDEs) of antioxidants [14,20,21,22,23]. Nevertheless, they are sometimes inconsistent with each other. Indeed, such data are basis set and solvent dependent. It is then crucial to have a reliable method that can accurately predict the BDEs of a large scope of phenols and build a predictive scale of their antioxidant power, supported by experimental data.

In this paper, we determine the BDEs of 70 natural (i.e., tocopherols, derivatives of hydroxybenzoic and cinnamic acids, flavonols, flavones, flavanonols, flavanones, isoflavones, flavanols, stilbenes, lignans, and coumarins) and synthetic (i.e., 2,6-di*-tert*-butyl-4-methylphenol (BHT), 3-*tert*-butyl-4-hydroxyanisol (BHA), *tert*-butylhydroquinone (TBHQ), and propyl gallate (PG)) antioxidants by density functional theory (DFT) calculation. The method is referred to as B3LYP/6-311++G(2d,2p)//B3LYP/6-311G(d,p) and allows the calculation of accurate BDEs in relative short time. On the other hand, kinetic rate constants and number of hydrogen atoms released per molecule of phenol have also been measured by monitoring the reaction of phenols with DPPH^•^ radical. The comparison of the results obtained with those two complementary methods allows highlighting the most promising antioxidants. Finally, the antioxidant effectiveness of the best candidates has been assessed under more realistic conditions by following during the oxidation process the absorption of oxygen by fatty acid methyl esters (FAMEs) of linseed oil containing 0.5 mmol·L^−1^ of antioxidant.

## 2. Results

### 2.1. Bond Dissociation Enthalpies (BDE) of 70 Phenolic Antioxidants

All of the (poly)phenols studied in this work are gathered by families in Table 1 and are classified from the lowest BDE to the highest BDE. The antioxidant power of 10 synthetic antioxidants, four tocopherols, eight hydroxybenzoic and eight hydroxycinnamic acids derivatives, 13 flavonols, two flavones, two flavanonols, four flavanones, three isoflavones, three catechins, two stilbenes, eugenol and isoeugenol, three phenols found in olive oil, one lignan, three coumarins, carnosic acid and carnosol are studied by DFT calculation. BDEs of all the O–H sites for each molecule have been calculated and results are described in supplementary materials (Table S1).

Table 2 summarizes the calculated BDEs by the B3LYP/6-311++G(2d,2p)//B3LYP/6-311G(d,p) method for the 70 phenols. Literature values are given in bracket and compared with our own values in supplementary materials (Figures S2–S4).

Because of toxicity, some efficient antioxidants still used for polymers are no longer tolerated in food products. In recent years, the toxicity of BHT **7** and BHA **5** have extensively been studied and they are now very controversial [38]. Consequently, their ban in the near future is expected. One of the current alternatives is the natural (poly)phenols. BDEs of synthetic phenols are in the following order: 5-*tert*-butylpyrigallol **1** < pyrogallol **2** < hydroxyquinol **3** < propyl gallate **4** < BHA **5** < 4-*tert*-butylcatechol **6** < BHT **7** < TBHQ **8** < *o*-*tert*-butyl-p-cresol **9** < phloroglucinol **10**.

Tocopherols are monophenolic compounds derived from chromanol which are very soluble in oils making α-tocopherol **11** the most important antioxidant in edible fats and oils [39]. These phenols are frequently found in vegetable oils especially soybean, sunflower and palm oils. The four derivatives of tocopherol are distinguishable by the number and the position of the methyl substituents, which impact the BDEs. α-Tocopherol **11** has the lowest BDE compared to the β-, γ- and δ-tocopherols.

Phenolic acids are another important class of antioxidants ubiquitous in food plants (i.e., fruit, vegetable, and coffee) [40]. There are simple phenolic acids based on hydroxybenzoic and hydroxycinnamic acids. BDEs of hydroxybenzoic acids are in the following order: Gallic acid **15** < protocatechuic acid **16** < syringic acid **17** < ellagic acid **18** < gentisic acid **19** < vanillic acid **20** < 4-hydroxybenzoic acid (PHBA) **21** < salicylic acid **22**. Ellagic acid **18** is a particular combination of two molecules of Gallic acid and has a BDE of 78.4 kcal·mol^−1^. Furthermore, the hydroxycinnamic acid with the lowest BDE is rosmarinic acid **23**. It is then followed by caffeic acid **24**, chlorogenic acid **25**, and sinapic acid **26**. The others derivatives of hydroxycinnamic acids have highest BDEs (BDE > 80 kcal·mol^−1^).

**Figure 1:**
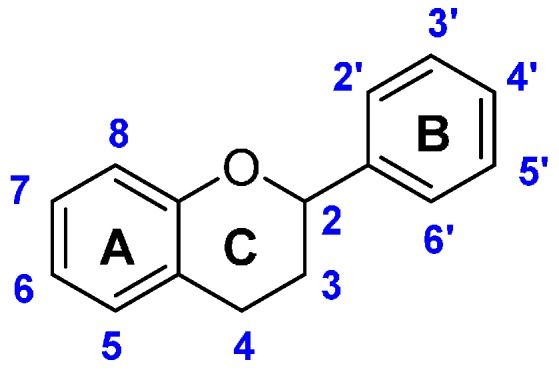
General chemical structure of flavonoids.

The class of flavonoids gathers more than 4000 different polyphenols found in leaves, stems, roots, fruits or seeds [41]. Their general chemical structure contains three rings A, B and C (Figure 1).

The presence of carbonyls, double bonds and hydroxyl groups on the pyranyl ring C divides the flavonoids into different subclasses called flavonols, flavones, flavanonol, flavonones, isoflavone and flavanols. Substitution of A and B rings distinguishes the different phenolic antioxidants of each subclasses. The antioxidant activity of flavonoids depends on various factors [41]: (i) the metal-chelating potential that is strongly dependent on the arrangement of hydroxyls and carbonyl group around the molecule [42]; (ii) the presence of electron-donating substituents; and (iii) their ability to delocalize the unpaired electron leading to the formation of stable phenoxyl radical. Moreover, it has been shown that the phenolic ring B is the most active cycle [43].

Flavonols (i.e., gossypetin **31**, myricetin **32**, quercetin **34** and morin **41**) have the 3-hydroxyflavone backbone which includes double bond and hydroxyl group on the pyranyl ring C. The flavonols with the lowest BDE are gossypetin **31** (66.6 kcal·mol^−1^), myricetin **32** (67.4 kcal·mol^−1^) and quercetin **34** (71.8 kcal·mol^−1^). BDEs of flavonols depend on the number of hydroxyl groups and their location on the structure of flavonols, which is discussed later.

Flavones such as luteolin **44** and apigenin **45** are mainly found in cereals and herbs. They have the same chemical structure as flavonols without the hydroxyl group on the pyranyl ring C.

Flavanonols (i.e., taxifolin **46** and aromadedrin **47**) and flavanones (i.e., eriodictyol **48**, homoeriodictyol **49**, hesperetin **50** and naringenin **51**) are other classes of flavonoids. They have the same chemical structure as flavonols but without the double bond on the pyranyl ring C and taxifolin **46** has a lower BDE than aromadedrin **47**. Flavonones do not have double bond and hydroxyl group on the pyranyl site C. The flavanone with the lowest BDE is eriodictyol **48** (73.6 kcal·mol^−1^).

Isoflavones (i.e., glycetin **52**, genistein **53** and daidzein **54**) are also studied. They are similar with flavones except that the B ring is bound to the C(3) position instead of the C(2). The three isoflavones studied have almost the same BDE. The OH group involved is located on the carbon C(4’).

Finally, the last class of flavonoid studied is catechins, also called flavanols, which are abundant in tea (i.e., epigallocatechin gallate **55**, gallocatechin **56** and catechin **57**). The catechin with the lowest BDE is epigallocatechin gallate **55** (66.5 kcal·mol^−1^).

The two investigated stilbenes (i.e., piceatannol **58** and resveratrol **59**) are natural polyphenols present in many plants such as grapes. Piceatannol **58** differs from resveratrol **59** with an OH group at the C(3’) position which decreases the BDE.

Eugenol **61** is a phenol found in clove essence oil whereas isoeugenol **60** is present in ylang-ylang essential oil. The position of the double bond influences the BDE leading to a higher value for eugenol **61**.

Hydroxytyrosol **62**, catechol **63** and tyrosol **64** are antioxidants found in olive oil [44]. Hydroxytyrosol **62** is the phenol with the lowest BDE followed by catechol **63** and tyrosol **64** (81.0 kcal·mol^−1^).

Sesamol **65** is a lignan found in sesame oil. It is a potent antioxidant and antiflammatory agent in various oxidative systems [45]. Lignans are phenyl propanoids derivated from phenylalanine and include also sesamin, sesamolin, sesaminol and sesamolinol [39].

The main coumarin called aesculetin **67** is found in tonka bean. Methyl and phenyl substituents can be grafted at the C(4) position but they have no impact on BDEs.

Carnosol **69** and carnosic acid **70** are the two major components with rosmarinic acid **23** (already described) of rosemary extract (*Rosmarinus officinalis* L.) and are authorized in food in the form of extract [46]. For both compounds, same BDE (70.8 and 70.7 kcal·mol^−1^) was found.

### 2.2. Kinetic Rates of Hydrogen Transfer, Stoichiometric Numbersand Inhibition of FAMEs Linseed Oil Oxidation

Thirty-two phenols (**1**, **4**–**9**, **11**, **15**–**17**, **20**–**27**, **32**, **34**, **55**, **58**–**63**, **65**, **67**, **69** and **70**) have been selected to cover the different classes of antioxidants and confirm their antioxidant activity through the DPPH^•^ (2,2-diphenyl-1-picrylhydrazyl) test and during the oxidation of FAMEs linseed oil (RapidOxy^®^). All these experimental methods have already been described in our previous works [27,29,47].

#### 2.2.1. Kinetic Rates of Hydrogen Transfer

The DPPH^•^ test [48,49,50,51,52,53] is commonly used to evaluate the antioxidant power of phenolic compounds. DPPH^•^ is a stable radical with a maximum of absorption around 515–520 nm (purple). When antioxidants are mixed with this stable radical, there is a transfer of hydrogen from the antioxidant to the DPPH^•^ radical which becomes yellow (Equation (7)) [47]. Thus, it is easy to follow the hydrogen transfer by UV-visible spectrometry. Toluene has been chosen as a solvent because it is inert towards radical reactions and cannot create hydrogen bonding. Indeed, the polarity of the solvent can drastically change the reactivity of polar antioxidants. The mechanism involved in this apolar aprotic solvent is called hydrogen atom transfer (HAT), which is the opposite of the sequential proton loss electron transfer (SPLET) mechanism that takes place in polar solvents [29].

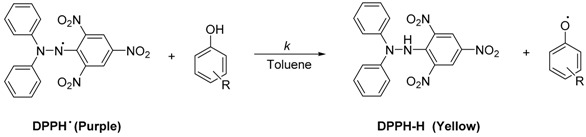
(7)

**Figure 2:**
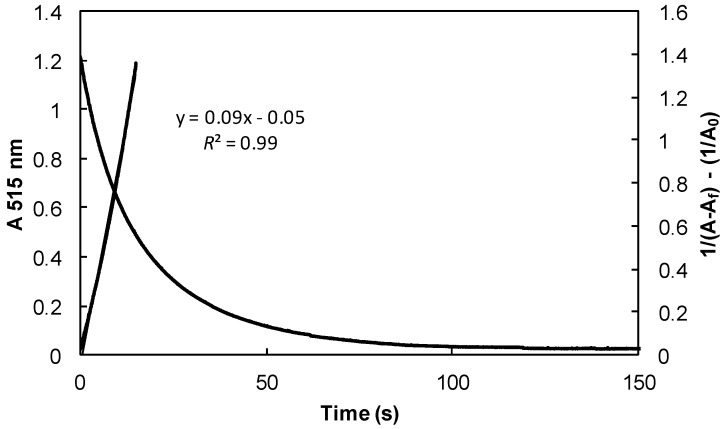
Evolution of the absorbance of DPPH^•^ radical at 515 nm (1.25 × 10^−4^ mol·L^−1^) in the presence of hydroxytyrosol **62** (1.25 × 10^−4^ mol·L^−1^) in toluene at 20 °C. Linearization curve according to second order kinetics (SOK) (Equation (8)).

Kinetic rates (*k*) are obtained using either pseudo-first order kinetics (FOK) or second order kinetics (SOK) depending on the reactivity of the phenol under study [29,47]. Reactive phenols (**1**, **4**, **5**, **6**, **8**, **11**, **17**, **20**, **26**, **27**, **60**, **61**, **62**, **63**, **65**, **69** and **70**) were mixed with DPPH^•^ in stoichiometric amount leading to SOK (Equation (8)) [47]. Figure 2 shows an example of SOK reaction with hydroxytyrosol **62** in toluene. Kinetic rate constant is determined during the first 20 seconds of the reaction and only takes into account the reaction of phenolic hydrogen with DPPH^•^.

(8) $$\frac{1}{\left(A\;-\;A_{f}\right)}\;=\;\frac{1}{\left(A_{0}\;-\;A_{f}\right)}\;-\;\frac{k}{\left(\varepsilon \;-\;\varepsilon \prime \right)}\;t$$

**Figure 3:**
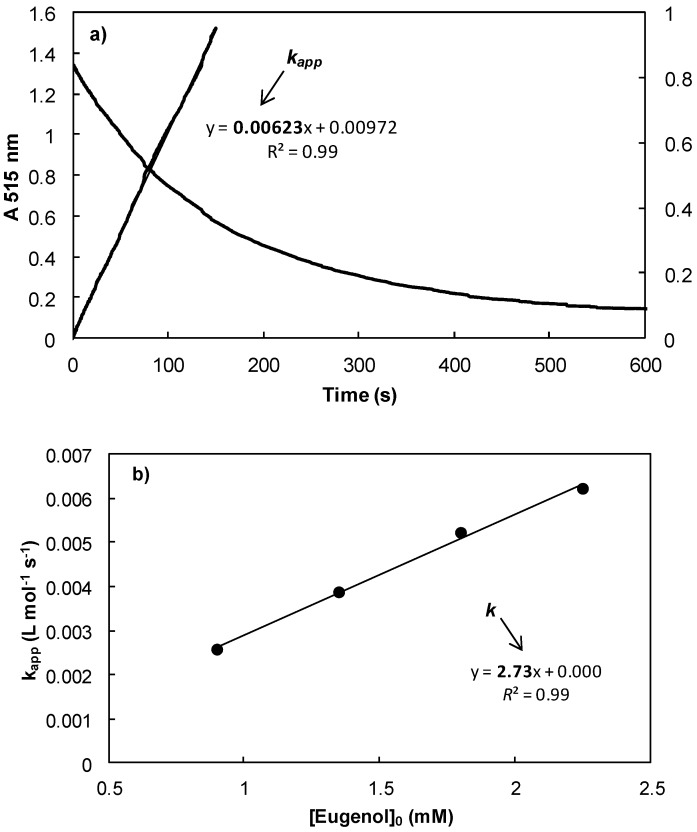
(**a**) Change in absorbance at 515 nm of a solution of DPPH^•^ (1.25 × 10^−4^ M) in the presence of an excess of eugenol **61** (2.25 × 10^−3^ M) in toluene, linearization of the logarithm of the absorbance using the final pseudo-first order kinetics (FOK) (Equation (9)) as a function time; and (**b**) regression constants of apparent kinetic rates as a function of initial concentrations of eugenol **61**.

On the other hand, weakly reactive phenols (**7**, **9**, **20** and **61**) are introduced in excess at different concentrations with respect to DPPH^•^. Under these conditions, [ArOH]_t_ ≈ [ArOH]_0_ and a pseudo-first order kinetics (FOK) describe the system (Equation (9)) [47]. Figure 3 shows the FOK reaction with eugenol **61** in toluene.

(9) $$Ln\;\frac{\left(A\;-A_{f}\right)}{\left(A_{0}\;-\;A_{f}\right)}\;=\;-\;k\;{\left[\text{ArOH}\right]}_{0}\;t$$

To confirm that pseudo-first order kinetic (FOK) and second order kinetic (SOK) give the same results for the same phenolic antioxidants, vanillic acid **20** and eugenol **61** were studied using both conditions leading to similar rate constants *k*. Results are gathered in Table 3.

#### 2.2.2. Stoichiometric Number (σ_exp_)

The second parameter highlighted by the DPPH^•^ test is the number of hydrogen transferred from the phenol to the stable radical called the stoichiometric number (σ_exp_). It is obtained via the final absorbance reached by DPPH^•^ in the presence of a large excess of DPPH^•^ with respect to the antioxidant concentration [54] (Equation (10)) [29,47].

(10) $$\sigma_{\exp}=\frac{{\left[{\text{DPPH}}^{•}\right]}_{0}-{\left[{\text{DPPH}}^{•}\right]}_{f}}{{\left[\text{ArOH}\right]}_{0}}=\frac{A_{0}-A_{f}}{(\varepsilon -\varepsilon \prime ){[\text{ArOH]}}_{0}}$$

The mechanism of interaction between DPPH^•^ radical and phenol takes place in two steps: (1) abstraction of the phenolic hydrogen; and (2) transfer of a second hydrogen or formation of dimers from the phenoxyl radical ArO^•^.

**Figure 4:**
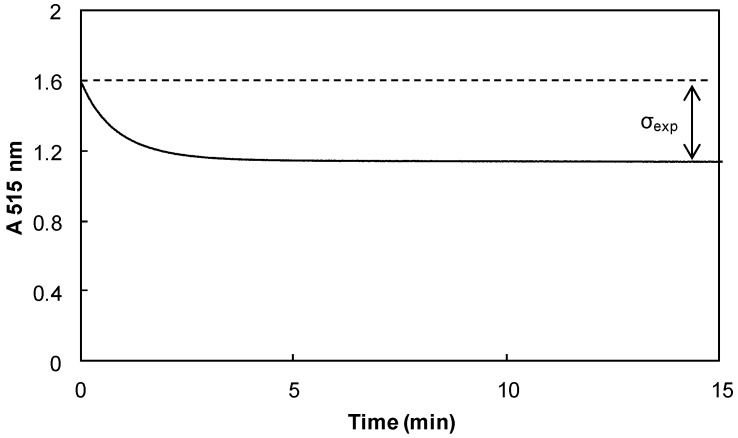
Evolution of the absorbance of DPPH^•^ radical (1.5 × 10^−4^ mol·L^−1^) at 515 nm in the presence of catechol **63** (2.07 × 10^−5^ mol·L^−1^) in toluene at 20 °C.

All of the stoichiometric numbers (σ_exp_) determined for the different phenols are summarized in Table 3. Toluene was replaced by ethyl acetate when antioxidants were not soluble. Figure 4 reports the result for catechol **63**.

#### 2.2.3. Inhibition of FAMEs Linseed Oil Oxidation

**Figure 5:**
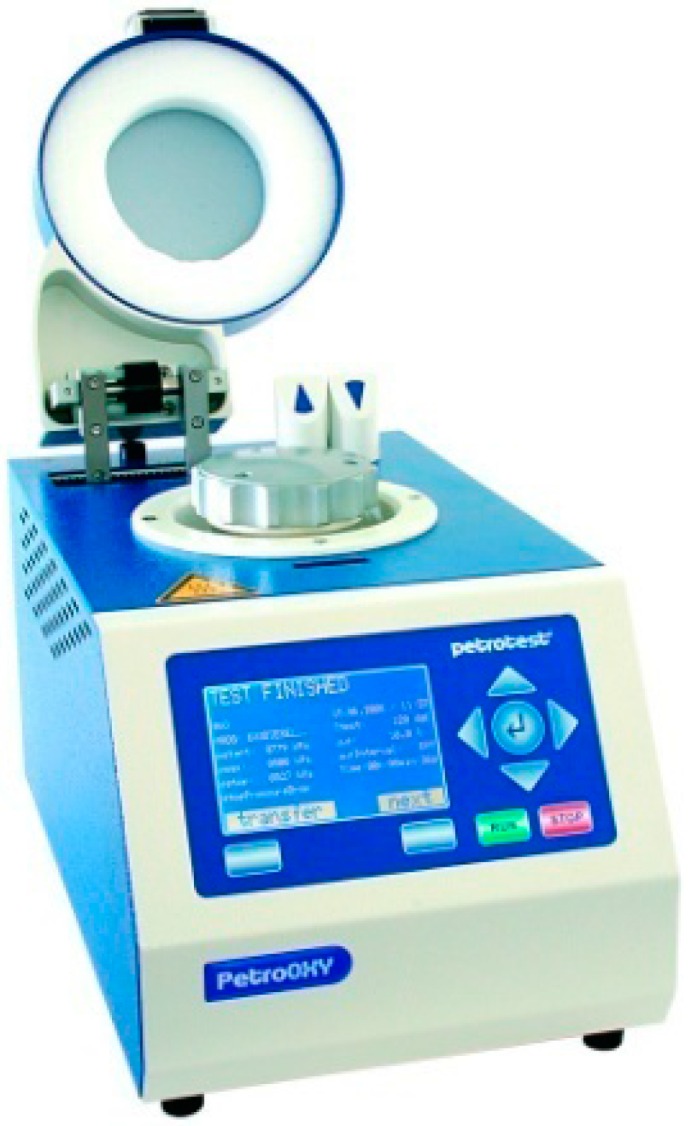
RapidOxy^®^ apparatus for measurement of oxygen consumption during the autoxidation process.

FAMEs of linseed oil were synthesized by transesterification [55]. Gas chromatography–mass spectrometry (GC-MS) analysis shows that they were composed of 5.3% methyl palmitate (C16:0), 5.3% methyl stearate (C18:0), 33.1% methyl oleate (C18:1), 11.2% methyl linoleate (C18:2) and 45.1% methyl linolenate (C18:3, ω-3). The autoxidation of omega-3 oils in the presence of the different phenols has been kinetically investigated by measuring the oxygen consumption via RapidOxy^®^ (Figure 5), which provides information on induction periods (IP) and oxidation rates (*R*_ox_) [47]. The efficiency of the antioxidants depends on their solubilization into the FAMEs. Indeed, a high solubility improves its protective effects of FAMEs against oxidation.

**Figure 6:**
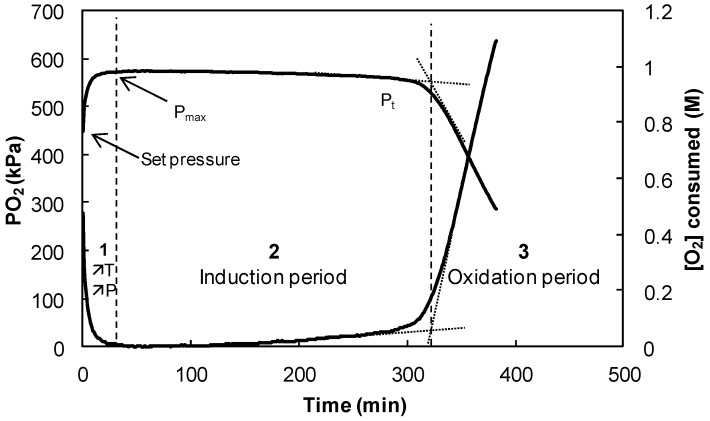
Evolution of the oxygen pressure (**left** axis) and concentration of oxygen consumed (**right** axis) during the oxidation of fatty acid methyl esters (FAMEs) in the presence of rosmarinic acid (5 × 10^−4^ mol·L^−1^) at 90 °C

As shown in Figure 6, the oxygen consumption during the oxidation process exhibits three steps: (i) the equilibration period corresponding to the increase of pressure following the increase of temperature after the achievement of the set pressure (450 kPa); (ii) the induction period defined by a slow decrease of the maximum pressure indicating that the antioxidant is effective; and (iii) the oxidation period characterized by a fast decrease of the oxygen consumption indicating the complete consumption of phenol which is no longer effective.

The evolution of the pressure was then converted to a concentration of oxygen consumed in the liquid phase ∆[O_2_]_t_ defined by Equation (11) [47], where *V*_tot_ and *V*_liq_ are the volumes of the cell and the FAMEs, respectively; *P*_max_ is the maximum pressure obtained a few minutes after heating the cell; and *P*_t_ is the pressure measured at a given time. Oxidation rate (*R*_ox_) is defined as the rate when oxygen is consumed in the presence of antioxidants. It corresponds to the slope of the trend curve of [O_2_] consumed during the induction period. The two important parameters (i.e., induction period IP and oxidation rate *R*_ox_) are compiled in Table 3.

(11) $$\Delta {\left[O_{2}\right]}_{t}=\frac{\left(P_{\max}-P_{t}\right)}{RT}\times \frac{\left(V_{\text{tot}}-V_{\text{liq}}\right)}{V_{\text{liq}}}$$

According to the kinetic rates constants obtained with the DPPH^•^ test, 5-*tert-*butyl-pyrogallol **1** (9480 M^−1^·s^−1^) and propyl gallate **4** (1240 M^−1^·s^−1^) are the most reactive synthetic phenols in toluene. With regard to natural phenolic antioxidants, α-tocopherol **11** exhibits the highest kinetic rate constants (2670 M^−1^·s^−1^) followed by carnosol **69** (1680 M^−1^·s^−1^), hydroxytyrosol **62** (1070 M^−1^·s^−1^) and carnosic acid **70** (640 M^−1^·s^−1^). Conversely, vanillic acid **20** (1.4 M^−1^·s^−1^), ferulic acid **27** (8.4 M^−1^·s^−1^) and eugenol **61** (3.9 M^−1^·s^−1^) are the least reactive phenols.

With regard to stoichiometric numbers (σ_exp_), three categories of antioxidants can be identified. First, there are the antioxidants capable of trapping more than three radical molecules (**4**, **15**, **23**, **32** and **55**, σ_exp_ ≥ 3). Then, other phenols transfer 2 or 3 hydrogens to DPPH^•^ radical (**1**, **5**, **7**, **8**, **11**, **16**, **24**, **25**, **27**, **34**, **58**, **61**, **62**, **63**, **65**, **67**, **69** and **70**, 2 ≤ σ_exp_ < 3). Finally, some compounds are not really active in the transfer of hydrogen since they trap less than two radicals per molecule of phenols (**17**, **26**, **59** and **60**, σ_exp_ < 2).

The comparison between induction periods observed for all phenolic antioxidants reveals four categories of phenols. First of all, epigallocatechin gallate **55** is by far the most reactive phenol with an induction period of about 500 min. It is followed by piceatannol **58** (IP = 313 min). These two compounds belong to category A including “extremely effective” antioxidants. Then phenols of category B (**1**, **6**, **23**, **32** and **70**) exhibit induction periods from 200 to 300 min and are considered “highly effective” antioxidants. Furthermore, phenols of category C (**4**, **5**, **7**, **11**, **15**, **24**, **25**, **34**, **62**, **63**, **65**, **67** and **69**) have an IP between 200 and 100 min and are considered as “moderately effective” antioxidants. Finally, phenols **8**, **9**, **16**, **17**, **20**, **21**, **26**, **27**, **59**, **60** and **61** of category D having an induction period lower than 100 min are “poorly effective” antioxidants.

During the oxidation of FAMEs linseed oil, epigallocatechin gallate **55** and 5-*tert-*butyl-pyrogallol **1** show the lowest oxidation rate (0.08 and 0.06 mM^−1^·s^−1^ respectively). Myricetin **32** also provides low oxidation rates of 0.11 mM^−1^·s^−1^, whereas, conversely, vanillic acid **20** does not reduce the oxidation rate (1.03 mM^−1^·s^−1^). It is noteworthy that α-tocopherol **11**, which is the phenol of reference, does not have the lowest oxidation rate (0.17 M^−1^·s^−1^).

## 3. Discussion

### 3.1. Bond Dissociation Enthalpies (BDE) of 70 Phenolic Antioxidants

It is commonly admitted in the literature that BDEs of phenols are strongly influenced by the number, nature and position of the substituents linked to the phenol ring [11,14,20,22,23,29,47,56,57,58]. Nevertheless, as the results are dependent on the method of calculation used, it is difficult to compare literature values. As an example, the BDE of apigenin **45** was found to be 75.6 kcal·mol^−1^ by Pérez-Gonzalez et al. [20] and 82.2 kcal·mol^−1^ by Leopoldini et al. [22]. Furthermore, BDEs of 5-*tert*-butylpyrogallol **1**, carnosol **69** and carnosic acid **70** have not been reported. Here, we use the B3LYP/6-311++G(2d,2p)//B3LYP/6-311G(d,p) method to calculate the BDE of the 70 phenols investigated. It is noteworthy that our theoretical results are globally consistent with those obtained by Leopoldini et al. (*R*^2^ = 0.98) [22], Li et al. (*R*^2^ = 0.96) [23] and Pérez-Gonzalez et al. (*R*^2^ = 0.97) [20] (see the good correlations in Figures S2–S4 in supplementary materials).

α-Tocopherol **11** exhibits a lower BDE than β-, γ- and δ-tocopherols. This low BDE (69.1 kcal·mol^−1^) results from different factors [27]: (1) the alkoxyl group in *p-*position; (2) the four alkyl substituents on the phenolic ring; (3) the molecular rigidity imposed by the pyran structure. Consequently, α-tocopherol **11** is expected to be the most powerful tocopherol.

**Figure 7:**
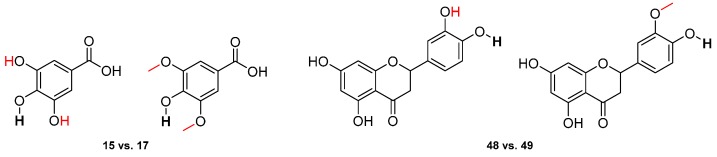
Comparison between gallic acid (**15**) and syringic acid (**17**) and between eriodictyol (**48**) with homoeriodictyol (**49**).

BDEs of hydroxybenzoic acid derivatives show that a substitution by two ortho-hydroxyl groups (Gallic acid, **15**) allows a much stronger decrease of the BDE than a substitution by two ortho-methoxy groups (syringic acid, **17**), which have BDEs of 70.2 and 78.1 kcal·mol^−1^, respectively. Moreover, this behavior is confirmed by comparing eriodictyol **48** (73.6 kcal·mol^−1^) and homoeriodictyol **49** (80.8 kcal·mol^−1^) (Figure 7). Nevertheless, ortho-carboxyl group (salicylic acid, **22**) drastically increases the BDE compared to that of phenol itself (82.2 kcal·mol^−1^).

**Figure 8:**
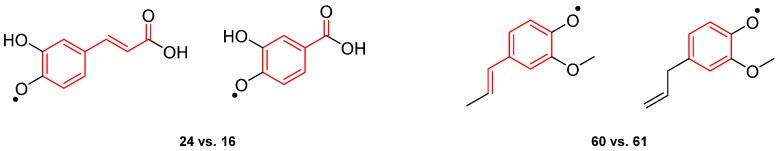
Comparison between caffeic acid (**24**) and protocatechuic acid (**16**) and between isoeugenol (**60**) and eugenol (**61**).

Thanks to a better delocalization of the unpaired electron for the phenolic radical, hydroxycinnamic acids have lower BDE than hydroxybenzoic acids. As a consequence, caffeic acid **24** (72.1 kcal·mol^−1^) should have a better antioxidant power compared to protocatechuic acid **16** (75.5 kcal·mol^−1^). Based on this argument, isoeugenol **60** has a lower BDE than eugenol **61** of ≈4 kcal·mol^−1^ (Figure 8).

BDE calculations of flavonoids highlight that the flavonol with the lowest BDE is gossypetin **31** (66.6 kcal·mol^−1^). As regards to its low BDE, it should be the most powerful flavonoid. The phenolic site involved is situated on the ring A as also demonstrated by Pérez-González et al. [20]. Except gossypetin **31**, the O–H group (R(4’) position) on the B ring is always the most reactive site [22]. However, flavonoids without this hydroxyl group (R(4’) position) are exceptions to this rule. As examples, kaempferide (**39**) and galangin (**43**) have their most hydroxyl reactive site on the ring C (C(3) position).

**Figure 9:**
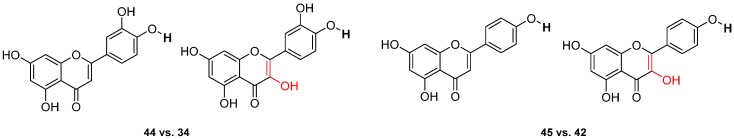
Comparison between flavones (**44** and **45**) and flavonols (**34** and **42**).

Flavones exhibit higher BDEs than flavonols. Indeed, luteolin **44** (73.1 kcal·mol^−1^) and apigenin **45** (82.1 kcal·mol^−1^) have higher BDE than quercetin **34** (71.8 kcal·mol^−1^) and kaempferol **42** (80.1 kcal·mol^−1^) respectively (Figure 9). This is due to the absence of OH group in the C ring. BDEs of O–H group for flavones in site R(4’) are about 10 kcal·mol^−1^ higher than for flavonols. Therefore, with equivalent substituents, flavones should be less reactive than flavonols through the HAT mechanism.

BDEs of isoflavones studied are close to that of apigenin **45** (82.1 kcal·mol^−1^) suggesting that the location of the ring B does not alter the hydrogen transfer. Therefore, the antioxidant power of flavones and isoflavone should be similar when they have the same number of hydroxyl groups on the ring B.

**Figure 10:**
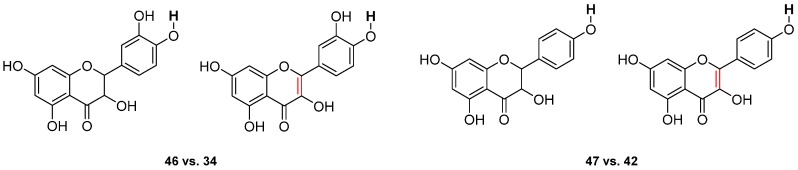
Comparison between flavanonols (**46** and **47**) and flavonols (**34** and **42**).

Flavanonols have higher BDEs than flavonols by comparing taxifolin **46** (73.2 kcal·mol^−1^) with quercetin **34** (71.8 kcal·mol^−1^) and kaempferol **42** (80.1 kcal·mol^−1^) with aromadedrin **47** (82.3 kcal·mol^−1^) (Figure 10). BDEs of O–H group for flavanonols in site R(4’) are about 2 kcal·mol^−1^ higher than for flavonols.

**Figure 11:**
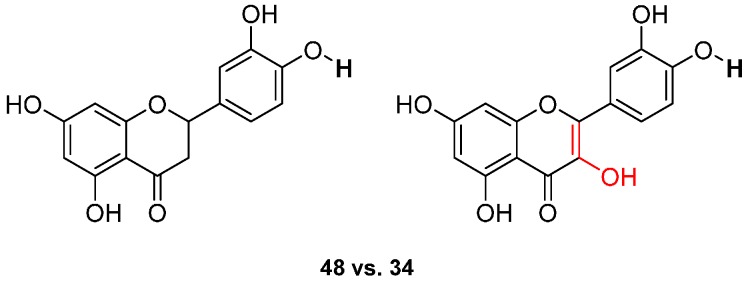
Comparison between flavanones (**48**) and flavonols (**34**).

Flavanones have higher BDEs than flavonols by comparing eriodictyol **48** (73.6 kcal·mol^−1^) and quercetin **34** (71.8 kcal·mol^−1^) (Figure 11). BDEs of the O-H group in site R(4’) is also about 2 kcal·mol^−1^ higher than for flavonols. That is a logical finding since the conjugation is broken due to the single bond. Therefore, the major effects are due to the neighboring groups.

Substitution of the phenolic ring by two ortho-hydroxyl groups improves the stability of the central hydroxyl group and also that of the phenolic radical leading to a drastic decrease of BDEs compared to phenol (82.2 kcal·mol^−1^) [11,47] (Equation (12)).

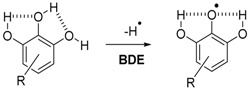
(12)

It is the case for epigallocatechin gallate **55** (66.5 kcal·mol^−1^), 5-*tert*-butylpyrogallol **1** (66.6 kcal·mol^−1^), myricetin **32** (67.4 kcal·mol^−1^), pyrogallol **2** (68.0 kcal·mol^−1^), propyl gallate **4** (69.6 kcal·mol^−1^) and Gallic acid **15** (70.2 kcal·mol^−1^).

Two ortho-hydroxyl groups (pyrogallol moieties) have a stronger impact on the decrease of the BDEs than only one ortho-hydroxyl function (catechol moieties). Indeed, pyrogallol structures (i.e., 5-*tert*-butylpyrogallol **1**, pyrogallol **2**, gallic acid **15**, myricetin **32** and gallocatechin **55**) have lower BDEs than their respective catechol compounds (i.e., 4-tert-butylcatechol **6**, catechol **63**, protocatechuic acid **16**, quercetin **34** and catechin **57**). Table 4 shows the comparison between these two types of antioxidants and highlights a systematic **Δ**BDE of ≈5 kcal·mol^−1^.

**Figure 12:**
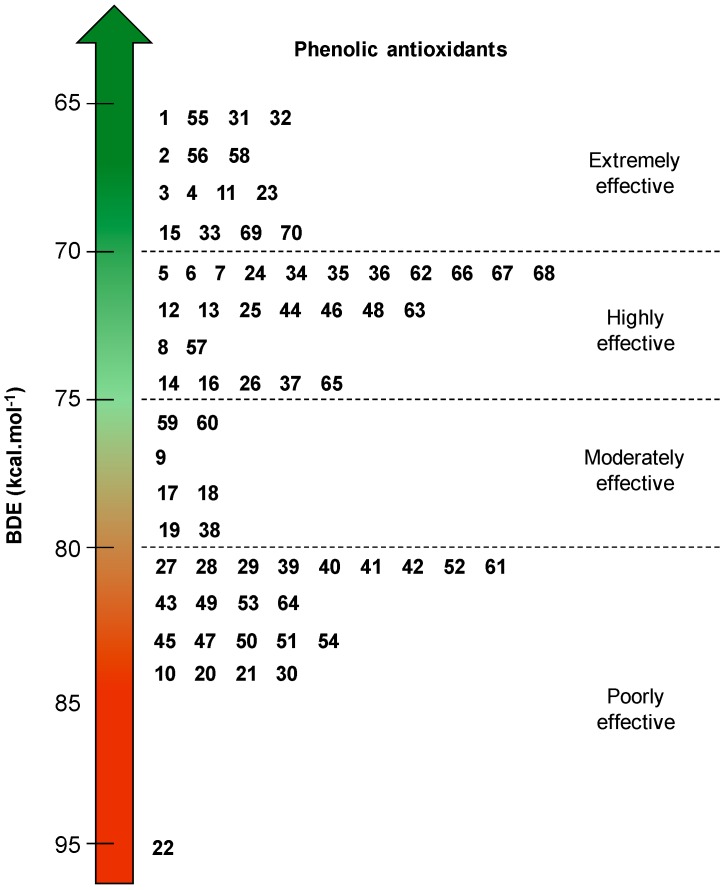
Scale of expected effectiveness of all the phenolic antioxidants studied (**1**–**70**) according to their BDE calculated with B3LYP/6-311++G(2d,2p)//B3LYP/6-311G(d,p) DFT method in vacuum.

Based on the BDEs of the studied phenols, a scale of predictive reactivity has been established from the lowest to the highest BDEs (Figure 12). It reveals four classes of antioxidants: (i) antioxidants with very low BDE from 65 to 70 kcal·mol^−1^; (ii) antioxidants with low BDE from 70 to 75 kcal·mol^−1^; (iii) antioxidants with medium BDE from 75 to 80 kcal·mol^−1^; and (iv) antioxidants with high BDE from 80 to 95 kcal·mol^−1^.

The antioxidants with the lowest BDEs are expected to have the best antioxidant power.

Very low BDEs (<70 kcal·mol^−1^) have been obtained for 5-*tert*-butylpyrogallol **1**, myricetin **32**, propyl gallate **4** and Gallic acid **15**, which are pyrogallol derivatives. This class contains also α-tocopherol **11** with a BDE of 69.1 kcal·mol^−1^. Rosmarinic acid **23**, carnosol **69** and carnosic acid **70** also exhibit a very low BDE. Indeed, they bear a catechol-type ring moiety, conjugated double bonds and alkyl substituents on the phenol rings which strongly contribute to lower the BDE.

Then, catechol **63** itself and catechol-based derivatives with flavonol structure (i.e., quercetin **34**), alkyl substituent (i.e., 4-*tert*-butyl-catechol **6**, hydroxytyrosol **62**) and conjugated double bonds (i.e., caffeic acid **24** and chlorogenic acid **25**) have low BDEs. Moreover, monophenols with OCH_3_ groups (BHA **5**), ortho-and para-alkyl substituents (BHT **7**) and substituted hydroquinone (TBHQ **8**) belong to this second class of antioxidants. Finally, there is also catechol-based derivative with electron-withdrawing group EWG (i.e., protocatechuic acid **16**) and monophenol with dioxolane moiety (i.e., sesamol **65**).

The category of antioxidants with medium BDE includes monophenols with OCH_3_ groups (i.e., syringic acid **17**, isoeugenol **60**), conjugated double bond (resveratrol **59**) and alkyl substituents (i.e., *o-tert-*butyl-*p*-cresol **9**).

Finally, vanillic acid **20**, PHBA **21**, ferulic acid **27**, eugenol **61** and tyrosol **64** have high BDEs and are expected to be poorly reactive considering the HAT mechanism. Moreover, the simplest structure of hydroxycinnamic acid derivatives (*o-*, *p-* and *m-*coumaric acids **28**, **29** and **30**) and phloroglucinol **10** (phenol with two OH groups in *meta* position) have the highest BDEs. Vanillic acid **20**, PHBA **21** and salicylic acid **22** have a higher BDE than phenol itself (kcal·mol^−1^) due to the effect of electron-withdrawing group (EWG).

We can conclude that a powerful antioxidant must have pyrogallol (i.e., Gallic acid **15**, myricetin **32**, epigallocatechin gallate **55** and gallocatechin **56**) or catechol moieties (i.e., rosmarinic acid **23**, carnosol **69** and carnosic acid **70**) conjugated with para-electron-donating substituents. They are the best natural alternatives to α-tocopherol **11** and synthetic phenolic antioxidants.

### 3.2. Kinetic Rates of Hydrogen Transfer, Stoichiometric Numbers and Inhibition of FAMEs Linseed Oil Oxidation

**Figure 13:**
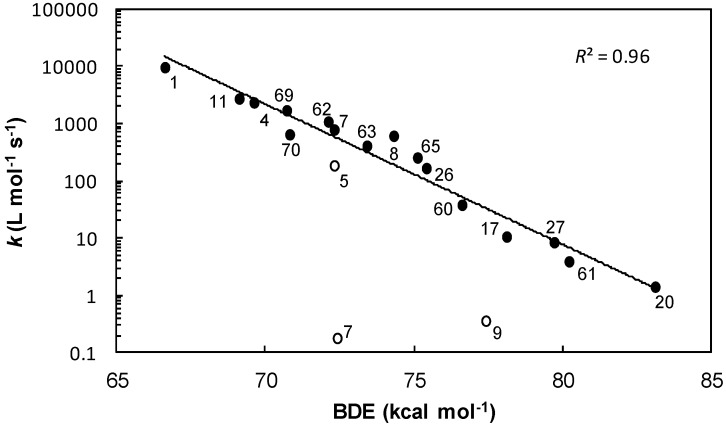
Logarithm of the rate constants (log k) for the reaction of phenolic antioxidants with DPPH^•^ (ο hindered phenols and ● other phenols) in toluene as a function of their BDEs calculated with the B3LYP/6-311++G(2d,2p)//B3LYP/6-311G(d,p) density functional theory (DFT) method.

The determination of the kinetic rates of hydrogen transfer for phenolic antioxidants is a way to experimentally confirm the antioxidant properties suggested by BDE calculations. The logarithm of the rate constants for the reaction of hydrogen transfer from phenol to the DPPH^•^ radical is very well correlated with the calculated BDE of phenols (*R*^2^ = 0.96) confirming that the radical HAT mechanism occurs in toluene [29,47] (Figure 13).

Nevertheless, due to the steric hindrance of the phenolic hydrogen, kinetic rates are sharply slowed down for BHA **5**, BHT **7** and *o-tert*-butyl-*p*-cresol **9**. Therefore, kinetic rates *k* are very low and do not follow the trend curve [27,47].

Log *k* decrease with increasing BDE has also been demonstrated by Foti et al. in heptane [34] and Marteau et al. in *m*-xylene [27]. Foti and co-workers have also proven that kinetics obtained with DPPH^•^ are correlated with the reaction of phenols with peroxyl radicals ROO^•^ [34,59]. Indeed, this test mimics the behavior of phenolic antioxidants during the inhibition of oils oxidation thanks to hydrogen transfer through a radical mechanism. Although the conditions and the method used to determine the kinetic rates of hydrogen transfer are different, our results obtained with the DPPH^•^ test are consistent with the literature and with our scale of reactivity based on BDEs.

**Figure 14:**
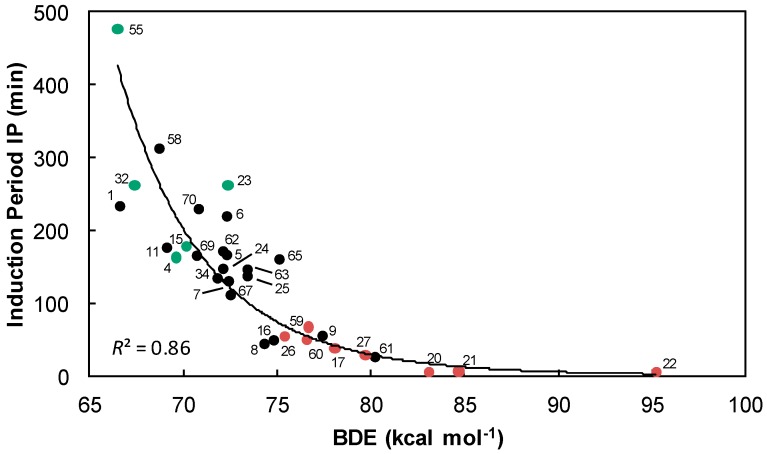
Induction periods (IP) as a function of the BDEs calculated with the B3LYP/6-311++G(2d,2p)//B3LYP/6-311G(d,p) DFT method, stoichiometric numbers are indicated by: ● σ_exp_ ≥ 3, ● 3 < σ_exp_ ≤ 2 and ● σ_exp_ < 2 and phenols are categorized as: extremely effective (**A**); highly effective (B); moderately effective (C); and poorly effective (D).

These theoretical (BDE) and kinetic (DPPH^•^ test) tools have allowed highlighting some promising effective antioxidants. Their potential antioxidant power has finally been evaluated against the oxidation of omega-3 oils derivatives (FAMEs). Figure 14 shows that the most efficient antioxidants are those with the lowest BDEs but no clear correlation between induction periods and BDEs could be obtained. There seems to be an exponential tendency (*R*^2^ = 0.86). Thereby, other parameters such as BDE influence the antioxidant power of phenols. The number of radicals trapped by molecule of antioxidant (σ_exp_) has an important impact on the inhibition of oxidation and influences the induction period. These stoichiometric numbers were obtained with the DPPH^•^ test and experiments point out that the most efficient antioxidants are those with the highest stoichiometric numbers (● σ_exp_ ≥ 3), whereas poorly effective phenols just trap fewer than two radicals per molecule (● σ_exp_ < 2).

The trend displayed is clear: highly effective antioxidants are polyphenols characterized by high stoichiometric numbers. As an example, epigallocatechin gallate **55** traps more than five radicals per molecule of antioxidant and delays the oxidation process of about 500 minutes. Conversely, poorly effective antioxidants are those with low stoichiometric numbers as for syringic acid **17**, isoeugenol **60** and sinapic acid **26**. They transfer too few hydrogen atoms to be efficient on the delayed action of the oxidation process. Basically, moderately effective antioxidants (**4**, **5**, **7**, **11**, **15**, **24**, **25**, **34**, **62**, **63**, **65**, **67** and **69**) trap two radicals per molecule.

Nevertheless, there are some exceptions: Gallic acid **15** and propyl gallate **4** have higher stoichiometric numbers compared to moderately effective antioxidants. The transfer of all the hydrogen atoms from these phenols is probably too low to be competitive with the oxidation of FAMEs. Moreover, phenols with equal or higher stoichiometric numbers as moderately effective antioxidants (σ_exp_ ≥ 2) could be characterized as poorly effective antioxidants as attested by their low induction periods (i.e., *o-tert*-butyl-*p*-cresol **9**, ferulic acid **27** and eugenol **61**). Finally, piceatannol **58**, which is also considered an extremely effective antioxidant, traps only two radicals per molecule of phenol.

**Figure 15:**
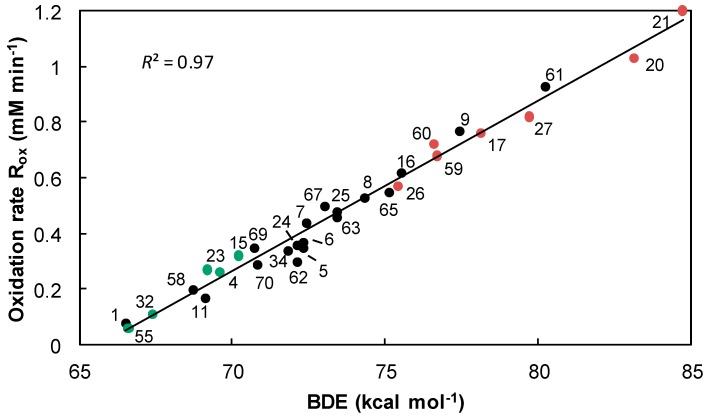
Oxidation rates as a function of the BDEs calculated with the B3LYP/6-311++G(2d,2p)//B3LYP/6-311G(d,p) DFT method, stoichiometric numbers are indicated by: ● σ_exp_ ≥ 3, **●** 3 < σ_exp_ ≤ 2 and ● σ_exp_ < 2.

Accordingly, the number of radicals trapped by one molecule of phenol (σ_exp_) is a crucial parameter for the protection of FAMEs against oxidation but the exceptions above-mentioned point out other essential factors as the BDE. Indeed, there is a very good correlation between oxidation rates (*R*_ox_) and BDEs (*R*^2^ = 0.97, Figure 15). We have previously described a correlation between BDEs and kinetic rates (DPPH^•^ test) involving a transfer of hydrogen according to the radical HAT mechanism. Thereby, we assume that the mechanism involved during the inhibition of oxidation by phenolic antioxidants is also a radical mechanism.

During the oxidation of FAMEs linseed oil, the lower the rate of oxygen consumption is, the more efficient the antioxidant is. Consequently, epigallocatechin gallate **55** and 5-*tert*-butyl-pyrogallol **1** are the most efficient antioxidants followed by myricetin **32**. Contrary to the observation made with the DPPH^•^ test, hindered phenols (i.e., BHA **5**, BHT **7** and *o-tert-*butyl-*p*-cresol **9**) are close to the correlation straight line. Their reactions with peroxyl radicals ROO^•^, which are less hindered than DPPH^•^, are not inhibited and they play their antioxidant role. Antioxidants with the lowest oxidation rate (*R*_ox_) are those with the highest stoichiometric numbers (● σ_exp_ ≥ 3) and conversely, poorly effective phenols are characterized by the highest R_ox_ and lowest stoichiometric numbers (● σ_exp_ < 2).

Consequently, the antioxidant power of phenols is determined by a combination of parameters: their BDE, the number of radicals trapped by one molecule of phenols and their ionization potential (IP) which has not been investigated here. As shown by Klein et al., the ionization potential of phenolic antioxidants has to be relatively high to be efficient in the protection of oxidized substrates [14]. Based on these characteristics, the four classes of antioxidants pointed out by the RapidOxy^®^ experiments can be explained as follows:

First of all, epigallocatechin gallate **55** is the most effective antioxidant due to its pyrogallol and galloyl moieties, which drastically decreases the BDE (66.5 kcal·mol^−1^) and increases the number of radicals traps by molecule (σ_exp_ = 5.4). Moreover, even if piceatannol **58** only traps two radicals, its low BDE allows decreasing the oxidation rate of FAMEs and strongly increasing the induction period.

Then, phenols of category B (**1**, **6**, **23**, **32** and **70**) are highly effective antioxidants. Pyrogallol structures (i.e., 5-*tert*-butyl-pyrogallol **1** and myricetin **32**) and catechol moieties (i.e., 4-*tert*-butyl-pyrogallol **6**, rosmarinic acid **23** and carnosic acid **70**) possessing ortho-, para- or conjugated electron-donating groups (EDG) have low BDEs and stoichiometric number (σ_exp_) between 2.0 and 3.0.

Furthermore, phenols of category C (**4**, **5**, **7**, **11**, **15**, **24**, **25**, **34**, **62**, **63**, **65**, **67** and **69**) are moderately effective antioxidants. Monophenols such as BHA **5**, BHT **7**, α-tocopherol **11** and sesamol **65** are able to transfer two hydrogens (σ_exp_ = 2). Moreover, all the catechol derivatives categorized as moderately effective antioxidants are just capable to transfer two hydrogens. Consequently, there is formation of ortho-quinone methide derivatives [47]. Gallic acid **15** and propyl gallate **4**, identified as exception by their higher stoichiometric number (5 and 3.9, respectively), do not transfer their hydrogen enough quickly and the oxidation takes place at a rate of 0.32 and 0.26 mM·min^−1^. Therefore, even if these antioxidants could transfer more than two hydrogens, they are not highly efficient for the protection of omega-3 oils.

Finally, phenols **8**, **9**, **10**, **16**, **17**, **20**, **21**, **26**, **27**, **59**, **60** and **61** belongs to category D and are considered as poorly effective antioxidants. Even if TBHQ **8** and protocatechuic acid **16** are catechol or hydroquinone derivatives capable to trap two radicals per molecule of phenol (σ_exp_ = 2), they are poorly reactive. Indeed, **16** reacts very slowly with the DPPH^•^ radical and **8** could be subjected to thermal decomposition, volatilization or absorption by the food leading to a decrease of its antioxidant power [60]. *o-tert-*butyl-*p*-cresol **9** transfers more than two radicals (σ_exp_ = 2.5) per molecule but its high BDE (77.4 kcal·mol^−1^) is not in favor of an easy transfer of hydrogens. The other phenols included in this last category are monophenolic compounds with low kinetic rates of hydrogen transfer, high BDEs and stoichiometric number lower than 2.0.

In conclusion, to estimate the efficiency of a phenolic antioxidant, it is necessary to combine both theoretical calculations of the BDEs and kinetic measurements (DPPH^•^ test) of the rate constants and stoichiometric numbers. Through a systematic study based on 70 phenols, several efficient antioxidants better than α-tocopherol could be identified allowing a deeper understanding of the structure/activity relationships. The main rules that can be drawn are that antioxidants with low BDE, high kinetic rate of hydrogen transfer (*k*) and high number of radicals trapped by one molecule of phenols (σ_exp_) are expected to be highly efficient providing that they act through the HAT mechanism. It appears that an efficient antioxidant should have pyrogallol or catechol moieties conjugated with para-electron-donating substituents. Apart from α-tocopherol **11**, epigallocatechin gallate **55**, piceatannol **58**, myricetin **32**, rosmarinic acid **23** and carnosic acid **70** are relevant alternatives to synthetic antioxidants such as propyl gallate **4**, BHA **5** and BHT **7** for the preservation of omega-3 oils.

## 4. Materials and Methods

### 4.1. Reagents

Catechol **63** (≥99%), 4-hydroxybenzoic acid **21** (PHBA, ≥99%), rosmarinic acid **23** (96%), quercetin **34** (≥98%), 2-*tert*-butyl-4-methylphenol **9** (99%), *tert*-butylhydroquinone **8** (TBHQ, 97%), 4-hydroxy-3-methoxybenzoic acid **20** (vanillic acid, 97%), sesamol **65** (98%), propyl gallate **4** (PG, ≥98%), isoeugenol **60** (98%), 3-*tert*-butyl-4-hydroxyanisol **5** (BHA, 98%), 3,4-dihydroxybenzoic acid **16** (protocatechuic acid, 97%), 2,6-di*-tert*butyl-4-methylphenol **7** (BHT, ≥99%), 3,4-dihydroxycinnamic acid **24** (caffeic acid, 97%), ferulic acid **27** (99%), α-tocopherol **11** (≥96%), were from Sigma-Aldrich (Lyon, France). 3,5-dimethoxy-4-hydroxycinnamic acid **26** (sinapic acid, 98%), 5-*tert-*butylpyrogallol **1** (97%), syringic acid **17** (≥98%), eugenol **61** (99%), 6,7-dihydroxycoumarin **67** (aesculetin, ≥98%), were from Alfa Aesar (Karlsruhe, Germany). 3,4-dihydroxyphenyl ethanol **62** (hydroxytyrosol), myricetin **32** (≥98%), chlorogenic acid **25** (≥95%) and carnosic acid **70** (≥95%) were from Cayman Chemical Company (Ann Arbor, MI, USA). Gallic acid **15** (≥95%) was from Acros Organics (Geel, Belgium) and 4-*tert-*butylpyrogallol **6** (≥98%) was from Merck (France). Resveratrol **59** (≥98%) was from Tokyo Chemical Industry (TCI, Zwijndrecht, Belgium) and carnosol **69** was from Chromadex (Irvine, CA, USA). Solvents were of the purest grade commercially available from Sigma-Aldrich. The 2,2-diphenyl-1-picrylhydrazyl (DPPH^•^) radical was purchased from Sigma-Aldrich and kept at a temperature lower than 5 °C. Aluminum oxide, basic, Brockmann I, for chromatography, 50–200 μm, 60 Å was from Acros Organics (Geel, Belgium). Refined linseed oil was from Vandeputte Group, Mouscron, Belgium. FAME mix GLC-10 containing palmitic acid methyl ester (C16:0), stearic acid methyl ester (C18:0), oleic acid methyl ester (C18:1), linoleic acid methyl ester (C18:2) and linolenic acid methyl ester (C18:3) was from Supelco (Saint-Quentin Fallavier, France).

### 4.2. Calculation of the Bond Dissociation Enthalpies BDEs (O–H)

The bond dissociation enthalpy or BDE is given by the difference between the enthalpy of the phenoxyl radical (plus that of the hydrogen atom) and that of the starting phenol as described by Equations (13) and (14).

(13) $$\text{ArO}-H\;+\;X^{•}\to {\text{ArO}}^{•}\;+\;X-H$$

(14) $$\text{BDE}(\text{ArO}-\text{H})\;=\;H_{f}^{0}({\text{ArO}}^{•})\;+\;H_{f}^{0}({\text{H}}^{•})\;-\;H_{f}^{0}(\text{ArO}-\text{H})$$

The geometries of all the parent molecules were firstly optimized using the PM3 method and then the DFT one by using the B3LYP/6-311G(d,p) basis set. The first method was used to speed up the convergence of the optimization by the second one. The zero-point energy (ZPE) is taken into account to correct the BDE values. Geometries from this method were used as inputs to the final energy B3LYP/6-311G++(2d,2p) calculation. For species having several conformers, all of them were investigated. The conformer with the lowest electronic energy is retained. For radicals, the optimization also used the PM3 step plus the final UB3LYP/6-311G(d,p) method. The zero-point energy (ZPE) is also taken into account to correct the BDE values. Geometries were then used as inputs to the final UB3LYP/6-311G++(2d,2p) calculation. Calculations were performed in toluene. The method is described as B3LYP/6-311++G(2d,2p)//B3LYP/6-311G(d,p).

### 4.3. Determination of the Rate Constants for Hydrogen Transfer from Phenols to DPPH^•^

Reactions of phenols with DPPH^•^ are operating in toluene. Solutions of DPPH^•^ were prepared in toluene at a concentration of approximately 5 × 10^−3^ mol·L^−1^. For phenols **7**, **9**, **16**, **21** and **61**, solutions were prepared in toluene at a concentration varying from 6 × 10^−2^ to 2 × 10^−1^ mol·L^−1^. Typically, 200–500 μL of the phenol solutions were added to 500 μL of DPPH^•^ solution in a 50 mL glass reactor equipped with a UV fiber (from Varian equipped with a dip-probe; Varian, les Ulis, France) containing 20 mL of deoxygenated solvent maintained at 20 °C. The hydrogen transfer reaction from phenol to the DPPH^•^ radical was accompanied by a change in the UV-visible spectrum and was monitored at 515 nm with a Varian spectrophotometer (Cary 50, 10 pts·s^−1^). The loss of DPPH^•^ absorbance in the presence of an excess of phenol follows pseudo-first-order kinetics (FOK). The rate constants were determined for poorly reactive phenols **7**, **9**, **16**, **21** and **61** for at least four different phenol concentrations by plotting *k*_DPPH•_ versus [phenol]. In the case of other highly/moderately reactive phenols, the reaction with the DPPH^•^ radical is very fast and the rate constant were determined by using stoichiometric conditions considering second order kinetics (SOK). For these phenols, solutions were prepared in toluene at a concentration of approximately 5 × 10^−3^ mol·L^−1^. Equipment for UV-visible analysis (Agilent, Les Ulis, France) and curve presenting the visualization of the lag time are presented in Figure S1. Values of the rate constants are given in the supplementary materials (Table S2). Under these conditions, ε and ε’ values are 11,788 L·mol^−1^·cm^−1^ and 24 L·mol^−1^·cm^−1^ for DPPH^•^ and DPPH-H respectively [29].

### 4.4. Determination of the Stoichiometric Number (σ_exp_) for the Reaction of Phenolic Antioxidants with DPPH^•^

Solutions of DPPH^•^ are prepared in toluene at a concentration of ca. 1.5 × 10^−4^ M by sonicating the mixture until all DPPH^•^ crystals were dissolved. The solutions are then maintained under argon at 20 °C. For phenols, solutions are also prepared in toluene at a concentration of 2.07 × 10^−3^ M by sonicating until all crystals are dissolved. Typically, 20 μL of the phenol solutions are added to 2.0 mL of a DPPH^•^ solution in a UV cell stirred and maintained at 20 °C. The absorbance change is monitored at 515 nm by using the UV-Visible Cary 60 (Agilent, Les Ulis, France) every seconds or minutes. Final (*A*_f_) and initial (*A*_0_) absorbances are used to determine the stoichiometric number (σ_exp_) according to Equation (15). Final absorbances are collected when constant values are reached during at least thirty minutes. Values of the stoichiometric numbers are summarized in Table 3 and detailed in the Supplementary Information (Table S3) [30].

(15) $$\sigma_{\text{exp}}=\frac{{\left[{\text{DPPH}}^{•}\right]}_{0}-{\left[{\text{DPPH}}^{•}\right]}_{f}}{{\left[\text{ArOH}\right]}_{0}}=\frac{A_{0}-A_{f}}{(\varepsilon -\varepsilon \prime ){\left[\text{ArOH}\right]}_{0}}$$

### 4.5. Synthesis of Antioxidant-Free Fatty Acid Methyl Esters (FAMEs) by Transesterification of Purified Linseed Oil

Linseed oil was beforehand purified 3 times by alumina column chromatography to reach very low concentration of antioxidants naturally present in neat oil. The transesterification reaction of triglycerides of purified linseed oil with methanol into fatty acid methyl esters (FAMEs) is given in Equation (16). One liter of methanol was introduced into a 2 L three-necked equipped with a condenser and a gas bubbling. Sodium (10 g, 2 equiv.) was introduced piece by piece under argon followed by purified linseed oil (200 g, 1 equiv.). The reaction was performed during 12 h under magnetic stirrer. FAMEs were extracted with 3 × 300 mL of petroleum ether. The combined organic phases were evaporated under pressure. Isolated FAMEs were stored at −20 °C.

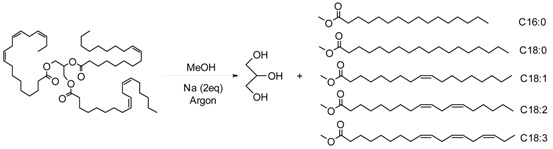
(16)

### 4.6. Analysis of FAMEs Linseed Oil by GC-MS

A Thermofisher (Courtaboeuf, France) GC Trace equipped with an AI 3000 injector connected to DSQ II simple quadrupole detector was used for the GC-MS analysis of FAMEs. Compound separation was achieved on a 30 m, DB5MS with 0.25 mm i.d. and 0.25 μm film thickness gas chromatographic column (J & W Scientific, Folsom, CA, USA). Carrier gas (ultra-pure helium) flow rate is 1.0 mL/min and the injector, the transfer line and the ions source were maintained at 250, 270 and 220 °C, respectively. The mass spectrometry (MS) detector was used in the electron ionization (EI) mode with an ionization voltage of 70 eV. The column was held at 130 °C for 0.5 min and then programmed at 0.3 °C·min^−1^ to 180 °C and maintained for 5 min. Then, the column was programmed at 3 °C·min^−1^ to 250 °C and maintained for 10 min. The compounds were injected in the Split mode with a ratio of 20. FAME mix GLC-10 (Sigma Aldrich, Lyon, France) was used to analyze and quantify the FAMEs composition.

### 4.7. Effect of the Phenolic Antioxidants on the Autoxidation of Fames Linseed Oil

Two-milliliter FAMEs of linseed oil were introduced into the RapidOxy cell (25 mL) at room temperature. One hundred microliters of phenol (**1**, **4**, **5**, **6**, **7**, **8**, **9**, **11**, **15**, **16**, **17**, **20**, **21**, **22**, **23**, **24**, **25**, **26**, **27**, **32**, **34**, **55**, **58**, **59**, **60**, **61**, **62**, **63**, **65**, **67**, **69** and **70**) solutions (10^−2^ mol·L^−1^) were then added to reach a final antioxidant concentration of 5 × 10^−4^ mol·L^−1^. Antioxidants are solubilized in ethyl acetate and an ultrasound bath is used to homogenize solutions. Few amount of ethanol could be firstly used to pre-solubilize antioxidants not soluble in ethyl acetate. The cell was the closed and heated up to the temperature set (90 °C) under a pure oxygen pressure of 450 kPa. The O_2_ consumption was followed by monitoring the O_2_ pressure. The experiment was ended when the pressure reached 50% of the maximum pressure. The pressure decrease was converted into a concentration of oxygen per volumes of the FAMEs solution. Values of the induction periods and oxidation rates are summarized in Table 3 and detailed in the Supplementary Information (Table S4).

## Figures and Tables

**Table 1:** Names and numbers of the polyphenols studied in this work.

N°	Name	N°	Name	N°	Name
	**Synthetic phenols**		**Flavonols**		**Catechins**
**1**	5-*Tert*-butylpyrogallol	**31**	Gossypetin	**55**	Epigallocatechin gallate
**2**	Pyrogallol	**32**	Myricetin	**56**	Gallocatechin
**3**	Hydroxyquinol	**33**	Azaleatin	**57**	Catechin
**4**	Propyl gallate	**34**	Quercetin		
**5**	BHA	**35**	Fisetin		**Stilbenes**
**6**	4-*Tert-*butylcatechol	**36**	Laricitrin	**58**	Piceatannol
**7**	BHT	**37**	Syringetin	**59**	Resveratrol
**8**	TBHQ	**38**	Rhamnazin		
**9**	*o-Tert*-butyl-*p*-cresol	**39**	Kaempferide		**Aromatic phenols**
**10**	Phloroglucinol	**40**	Isorhamnetin	**60**	Isoeugenol
		**41**	Morin	**61**	Eugenol
	**Tocopherols**	**42**	Kaempferol		
**11**	α-Tocopherol	**43**	Galagin		**Phenols from olive oil**
**12**	β-Tocopherol			**62**	Hydroxytyrosol
**13**	γ-Tocopherol		**Flavones**	**63**	Catechol
**14**	δ-Tocopherol	**44**	Luteolin	**64**	Tyrosol
		**45**	Apigenin		
	**Hydroxybenzoic acids**				**Lignans**
**15**	Gallic acid		**Flavanonols**	**65**	Sesamol
**16**	Protocatechuic acid	**46**	Taxifolin		
**17**	Syringic acid	**47**	Aromadedrin		**Coumarins**
**18**	Ellagic acid			**66**	Methylesculetin
**19**	Gentisic acid		**Flavanones**	**67**	Aesculetin
**20**	Vanillic acid	**48**	Eriodictyol	**68**	Nordalbergin
**21**	PHBA	**49**	Homoeriodictyol		
**22**	Salicylic acid	**50**	Hesperetin		**Carnosic acid derivatives**
		**51**	Naringenin	**69**	Carnosol
	**Hydroxycinnamic acids**			**70**	Carnosic acid
**23**	Rosmarinic acid		**Isoflavones**		
**24**	Caffeic acid	**52**	Glycitein		
**25**	Chlorogenic acid	**53**	Genistein		
**26**	Sinapic acid	**54**	Daidzein		
**27**	Ferulic acid				
**28**	*o*-Coumaric acid				
**29**	*p*-Coumaric acid				
**30**	*m*-Coumaric acid				

**Table 2:** Bond dissociation enthalpies (BDEs) for the phenolic antioxidants studied (**1**–**70**).

**Synthetic phenolic antioxidants**																		
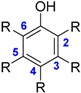																		
**N°**	**R(2)**	**R(3)**			**R(4)**							**R(5)**			**R(6)**			**BDE (kcal·mol^−1^)**
**1**	OH	H			C(CH_3_)_3_							H			OH			66.6 (nd)
**2**	OH	H			H							H			OH			68.0 (77.7 [24])
**3**	H	H			OH							H			OH			69.1 (70.4 [25])
**4**	OH	H			C(O)OC_3_H_7_							H			OH			69.6 (77.1 [23])
**5**	C(CH_3_)_3_	H			OCH_3_							H			H			72.3 (80.7 [23])
**6**	H	H			C(CH_3_)_3_							H			OH			72.3 (81.1 [26])
**7**	C(CH_3_)_3_	H			CH_3_							H			C(CH_3_)_3_			72.4 (79.9 [27])
**8**	H	H			OH							H			C(CH_3_)_3_			74.3 (76.9 [28])
**9**	H	H			CH_3_							H			C(CH_3_)_3_			77.4 (78.1 [29])
**10**	H	OH			H							OH			H			83.0 (87.7 [24])
**Tocopherols**																		
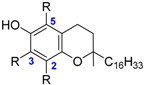																		
**N°**	**R(2)**					**R(3)**						**R(5)**						**BDE (kcal·mol^−1^)**
**11**	CH_3_					CH_3_						CH_3_						69.1 (71.7 [30])
**12**	CH_3_					H						CH_3_						73.4 (77.7 [11])
**13**	CH_3_					CH_3_						H						73.5 (78.2 [11])
**14**	CH_3_					H						H						75.4 (79.8 [11])
**Derivatives of hydroxybenzoic acids**																		
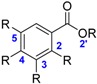																		
**N°**	**R(2)**	**R(3)**			**R(4)**				**R(5)**				**R(2’)**					**BDE (kcal·mol^−1^)**
**15**	H	OH			OH				OH				H					70.2 (72.2 [22])
**16**	H	OH			OH				H				H					75.5 (79.6 [24])
**17**	H	OCH_3_			OH				OCH_3_				H					78.1 (82.7 [31])
**18**	H	OH			OH				OC(O)-				-C_6_H(OH)_2_					78.4 (77.1 [32])
**19**	OH	H			H				OH				H					79.5 (80.0 [32])
**20**	H	OCH_3_			OH				H				H					83.1 (87.0 [31])
**21**	H	H			OH				H				H					84.7 (89.2 [24])
**22**	OH	H			H				H				H					95.2 (93.0 [24])
**Derivatives of hydroxycinnamic acids**																		
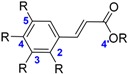																		
**N°**	**R(2)**	**R(3)**			**R(4)**				**R(5)**				**R(4’)**					**BDE (kcal·mol^−1^)**
**23**	H	OH			OH				H				C_9_O_4_H_10_					69.2 (75.3 [31])
**24**	H	OH			OH				H				H					72.1 (73.6 [22])
**25**	H	OH			OH				H				C_6_H_2_(OH)_3_CO_2_H					73.4 (78.7 [33])
**26**	H	OCH_3_			OH				OCH_3_				H					75.4 (81.2 [34])
**27**	H	OCH_3_			OH				H				H					79.7 (84.5 [24])
**28**	OH	H			H				H				H					80.1 (84.4 [24])
**29**	H	H			OH				H				H					80.5 (84.9 [24])
**30**	H	OH			H				H				H					84.4 (88.1 [35])
**Flavonols**																		
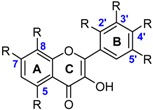																		
**N°**	**R(2’)**	**R(3’)**		**R(4’)**		**R(5’)**				**R(5)**		**R(7)**					**R(8)**	**BDE (kcal·mol^−1^)**
**31**	H	OH		OH		H				OH		OH					OH	66.6 (65.5 [20])
**32**	H	OH		OH		OH				OH		OH					H	67.4 (71.1 [36])
**33**	H	OH		OH		H				OCH_3_		OH					H	71.1 (66.1 [20])
**34**	H	OH		OH		H				OH		OH					H	71.8 (72.3 [22])
**35**	H	H		OH		OH				H		OH					H	72.3 (70.3 [36])
**36**	H	OCH_3_		OH		OH				OH		OH					H	72.5 (66.9 [20])
**37**	H	OCH_3_		OH		OCH_3_				OH		OH					H	75.7 (63.8 [20])
**38**	H	OCH_3_		OH		H				OH		OCH_3_					H	79.6 (65.2 [20])
**39**	H	H		OCH_3_		H				OH		OH					H	79.8 (73.8 [20])
**40**	H	OCH_3_		OH		H				OH		OH					H	79.8 (72.9 [20])
**41**	OH	H		OH		H				OH		OH					H	79.8 (76.9 [36])
**42**	H	H		OH		H				OH		OH					H	80.1 (80.9 [22])
**43**	H	H		H		H				OH		OH					H	81.2 (76.0 [36])
**Flavones**																		
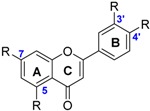																		
**N°**	**R(3’)**		**R(4’)**					**R(5)**						**R(7)**				**BDE (kcal·mol^−1^)**
**44**	OH		OH					OH						OH				73.1 (74.5 [22])
**45**	H		OH					OH						OH				82.1 (82.9 [22])
**Flavanonols**																		
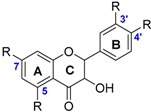																		
**N°**	**R(3’)**		**R(4’)**					**R(5)**						**R(7)**				**BDE (kcal·mol^−1^)**
**46**	OH		OH					OH						OH				73.2 (74.7 [22])
**47**	H		OH					OH						OH				82.3 (75.7 [20])
**Flavanones**																		
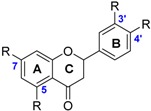																		
**N°**	**R(3’)**		**R(4’)**					**R(5)**						**R(7)**				**BDE (kcal·mol^−1^)**
**48**	OH		OH					OH						OH				73.6 (73.6 [36])
**49**	OCH_3_		OH					OH						OH				80.8 (75.1 [20])
**50**	OH		OCH_3_					OH						OH				82.2 (77.4 [36])
**51**	H		OH					OH						OH				82.4 (81.4 [36])
**Isoflavones**																		
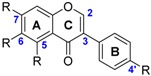																		
**N°**	**R(4’)**		**R(5)**					**R(6)**						**R(7)**				**BDE (kcal·mol^−1^)**
**52**	OH		H					OCH_3_						OH				80.1 (78.0 [36])
**53**	OH		OH					H						OH				81.0 (78.0 [36])
**54**	OH		H					H						OH				81.9 (78.3 [36])
**Catechins**																		
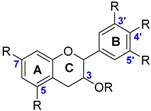																		
**N°**	**R(3’)**	**R(4’)**			**R(5’)**		**R(3)**				**R(5)**					**R(7)**		**BDE (kcal·mol^−1^)**
**55**	OH	OH			OH		C(O)C_6_H_2_(OH)_3_				OH					OH		66.5 (69.0 [36])
**56**	OH	OH			OH		H				OH					OH		68.5 (63.7 [20])
**57**	OH	OH			H		H				OH					OH		74.4 (74.2 [22])
**Stilbenes**																		
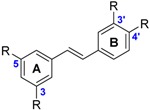																		
**N°**	**R(3‘)**		**R(4’)**					**R(3)**						**R(5)**				**BDE (kcal·mol^−1^)**
**58**	OH		OH					OH						OH				68.7 (62.9 [20])
**59**	H		OH					OH						OH				76.7 (70.3 [20])
**Eugenol and Isoeugenol**																		
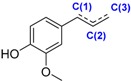																		
**N°**	**C(1)-C(2)**								**C(2)-C(3)**									**BDE (kcal·mol^−1^)**
**60**	-CH=CH-								-CH-CH_3_									76.6 (83.8 [27])
**61**	-CH_2_-CH-								-CH=CH_2_									80.2 (86.8 [27])
**Antioxidants in olive oil**																		
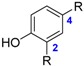																		
**N°**	**R(2)**								**R(4)**									**BDE (kcal·mol^−1^)**
**62**	OH								CH_2_CH_2_OH									72.1 (73.5 [22])
**63**	OH								H									73.4 (76.4 [24])
**64**	H								CH_2_CH_2_OH									81.0 (87.8 [23])
**Lignans**																		
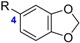																		
**N°**	**R(4)**																	**BDE (kcal·mol^−1^)**
**65**	OH																	75.1 (80.6 [34])
**Coumarins**																		
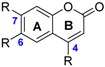																		
**N°**	**R(4)**					**R(6)**						**R(7)**						**BDE (kcal·mol^−1^)**
**66**	CH_3_					OH						OH						72.0 (72.1 [37])
**67**	H					OH						OH						72.5 (73.1 [37])
**68**	C_6_H_5_					OH						OH						72.6 (nd)
**Carnosol and carnosic acid**																		
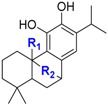																		
**N°**	**R(1)**								**R(2)**									**BDE (kcal·mol^−1^)**
**69**	/								-C(O)O-									70.7 (nd)
**70**	-CO_2_H								/									70.8 (nd)

nd: not determined.

**Table 3:** Rate constants (*k*) of hydrogen transfer from ArOH to DPPH^•^ in toluene at 20 °C, stoichiometric numbers of H atoms (σ_exp_) determined with an excess of DPPH^•^ in toluene at 20 °C, induction periods (IP) and oxidation rates (*R*_ox_) are determined by the RapidOxy^®^ experiments

N°	*k* (M^−1^·s^−1^) ^d^		Induction Period ^d^	Oxidation Rate ^d^	Stoichiometric Number
	SOK ^a^	FOK ^b^	IP (min)	*R*_ox_ (mM·min^−1^)	σ_exp_
**0 ^e^**	/	/	0	1.23	/
**1**	9480		234	0.06	2.1
**4**	1240		162	0.26	3.9
**5**	184		167	0.35	2.0
**6**	776		220	0.37	2.5
**7**		0.18	131	0.44	2.0
**8**	600		45	0.53	2.0
**9**		0.36	56	0.77	2.5
**11**	2690		177	0.17	2.0
**15**	ns		178	0.32	5.0 ^c^
**16**	ns		50	0.62	1.9 ^c^
**17**	10.6		37	0.76	1.1
**20**	1.4	1.0	5	1.03	0 ^c^
**21**	ns		6	1.20	0 ^c^
**23**	ns		262	0.27	4.1 ^c^
**24**	ns		148	0.36	2.0 ^c^
**25**	ns		138	0.48	1.9 ^c^
**26**	165		54	0.57	1.4 ^c^
**27**	8.4		28	0.82	1.8
**32**	ns		262	0.11	3.4 ^c^
**34**	ns		135	0.34	1.9 ^c^
**55**	ns		476	0.08	5.4 ^c^
**58**	ns		313	0.29	2.0 ^c^
**59**	ns		67	0.68	0.9 ^c^
**60**	38		49	0.72	0.9
**61**	3.9	2.7	27	0.93	2.1
**62**	1070		172	0.30	2.0
**63**	400		147	0.46	1.9
**65**	250		161	0.55	2.1
**67**	ns		112	0.50	2.1
**69**	1680		166	0.35	1.9
**70**	640		230	0.29	2.0

^a^, SOK: Second Order Kinetics ([DPPH^•^]_0_ = [ArOH]_0_); ^b^, FOK: pseudo-First Order Kinetics ([ArOH]_0_ >> [DPPH^•^]_0_); ^c^, ethyl acetate is used as a solvent; ^d^, average on three values, ns: not soluble; ^e^, blank with no antioxidant.

**Table 4:** BDEs comparison between pyrogallol (**15**, **1**, **2**, **32** and **55**) and catechol (**16**, **6**, **63**, **34** and **57**) moieties.

Pyrogallol Moieties		Catechol Moieties		ΔBDE (kcal·mol^−1^)
N°	BDE (kcal·mol^−1^)	N°	BDE (kcal·mol^−1^)	
**1**	66.6	**6**	72.3	5.7
**2**	68.0	**63**	73.4	5.4
**15**	70.2	**16**	75.5	5.3
**32**	67.4	**34**	71.8	4.4
**55**	68.5	**57**	74.4	5.9

ΔBDE = BDE (catechol) − BDE (pyrogallol).

## Supplementary Materials

Supplementary materials can be found at http://www.mdpi.com/1422-0067/17/8/1220/s1.

## Abbreviations

BHT	Butylated hydroxytoluene
BDE	Bond dissociation enthalpy
BHA	Butylated hydroxyanisole
DFT	Density functional theory
TBHQ	*tert-*Butylhydroquinone
EDG	Electron-donating group
PG	Propyl gallate
EWG	Electron-withdrawing group
FOK	Pseudo-first-order kinetic
LOO^•^	Lipid peroxyl radical
SOK	Second order kinetic
LOOH	Lipid hydroperoxide
DPPH	2,2-Diphenyl-1-picrylhydrazyl
HAT	Hydrogen atom transfer
FAMEs	Fatty acid methyl esters
IP	Induction period
LH	Unsaturated lipids
*R*_ox_	Oxidation rate

## References

[1] Burr M.L. (2000). Lessons from the story of n-3 fatty acids. Am. J. Clin. Nutr..

[2] Kryzhanovskii S.A., Vititnova M.B. (2009). Ω-3 polyunsaturated fatty acids and the cardiovascular system. Hum. Physiol..

[3] Kanner J., Rosenthal I. (1992). An assessment of lipid oxidation in foods. Pure Appl. Chem..

[4] Miyamoto S., Martinez G.R., Medeiros M.H.G., di Mascio P. (2003). Singlet molecular oxygen generated from lipid hydroperoxides by the russell mechanism: Studies using 18(O)-labeled linoleic acid hydroperoxide and monomol light emission measurements. J. Am. Chem. Soc..

[5] Neff W.E., Frankel E.N. (1984). Photosensitized oxidation of methyl linolenate monohydroperoxides: Hydroperoxy cyclic peroxides, dihydroperoxides and hydroperoxy bis(cyclic peroxide)s. Lipids.

[6] Halliwell B., Aeschbach R., Löliger J., Aruoma O.I. (1995). The characterization of antioxidants. Food Chem. Toxicol..

[7] Zhang H.-Y., Sun Y.-M., Wang X.-L. (2003). Substituent effects on o-h bond dissociation enthalpies and ionization potentials of catechols: A dft study and its implications in the rational design of phenolic antioxidants and elucidation of structure-activity relationships for flavonoid antioxidants. Chem. Eur. J..

[8] Van Acker S.A.B.E., Koymans L.M.H., Bast A. (1993). Molecular pharmacology of vitamin e: Structural aspects of antioxidant activity. Free Radical Biol. Med..

[9] Zhang H.-Y. (1999). Theoretical methods used in elucidating activity differences of phenolic antioxidants. J. Am. Oil Chem. Soc..

[10] Wright J.S., Carpenter D.J., McKay D.J., Ingold K.U. (1997). Theoretical calculation of substituent effects on the o-h bond strength of phenolic antioxidants related to vitamin e. J. Am. Chem. Soc..

[11] Wright J.S., Johnson E.R., DiLabio G.A. (2001). Predicting the activity of phenolic antioxidants:  Theoretical method, analysis of substituent effects, and application to major families of antioxidants. J. Am. Chem. Soc..

[12] Zhang H.-Y. (1998). Selection of theoretical parameter characterizing scavenging activity of antioxidants on free radicals. J. Am. Oil Chem. Soc..

[13] Zhang H.-Y. (2003). On the o-h bond dissociation enthalpy of catechol. New J. Chem..

[14] Klein E., Lukes V. (2006). Study of gas-phase O–H bond dissociation enthalpies and ionization potentials of substituted phenols-applicability of ab initio and DFT/B3LYP methods. Chem. Phys..

[15] Klein E., Lukes V., Cibulkova Z., Polovkova J. (2006). Study of N–H, O–H, and S–H bond dissociation enthalpies and ionization potentials of substituted anilines, phenols, and thiophenols. J. Mol. Struct. Theochem.

[16] Pratt D.A., DiLabio G.A., Brigati G., Pedulli G.F., Valgimigli L. (2001). 5-Pyrimidinols: Novel chain-breaking antioxidants more effective than phenols. J. Am. Chem. Soc..

[17] Bowry V.W., Ingold K.U. (1999). The unexpected role of vitamin E (α-tocopherol) in the peroxidation of human low-density lipoprotein. Acc. Chem. Res..

[18] Gotoh N., Noguchi N., Tsuchiya J., Morita K., Sakai H., Shimasaki H., Niki E. (1996). Inhibition of oxidation of low density lipoprotein by vitamin e and related compounds. Free Radic. Res..

[19] Noguchi N., Okimoto Y., Tsuchiya J., Cynshi O., Kodama T., Niki E. (1997). Inhibition of oxidation of low-density lipoprotein by a novel antioxidant, BO-653, prepared by theoretical design. Arch. Biochem. Biophys..

[20] Perez-Gonzalez A., Rebollar-Zepeda A.M., Leon-Carmona J.R., Galano A. (2012). Reactivity indexes and O–H bond dissociation energies of a large series of polyphenols: Implications for their free radical scavenging activity. J. Mex. Chem. Soc..

[21] Bakalbassis E.G., Lithoxoidou A.T., Vafiadis A.P. (2003). Theoretical calculation of accurate absolute and relative gas- and liquid-phase O–H bond dissociation enthalpies of 2-mono- and 2,6-disubstituted phenols, using dft/b3lyp. J. Phys. Chem. A.

[22] Leopoldini M., Russo N., Toscano M. (2010). The molecular basis of working mechanism of natural polyphenolic antioxidants. Food Chem..

[23] Li M.-J., Liu L., Fu Y., Guo Q.-X. (2007). Accurate bond dissociation enthalpies of popular antioxidants predicted by the oniom-g3b3 method. J. Mol. Struct. Theochem.

[24] Hoelz L.V.B., Horta B.A.C., Araujo J.Q., Albuquerque M.G., Bicca de Alencastro R., da Silva J.F.M. (2010). Quantitative structure-activity relationships of antioxidant phenolic compounds. J. Chem. Pharm. Res..

[25] Thavasi V., Leong L.P., Bettens R.P.A. (2006). Investigation of the influence of hydroxy groups on the radical scavenging ability of polyphenols. J. Phys. Chem. A.

[26] Amorati R., Ferroni F., Lucarini M., Pedulli G.F., Valgimigli L. (2002). A quantitative approach to the recycling of α-tocopherol by coantioxidants. J. Org. Chem..

[27] Marteau C., Nardello-Rataj V., Favier D., Aubry J.M. (2013). Dual role of phenols as fragrances and antioxidants: Mechanism, kinetics and drastic solvent effect. Flavour Frag. J..

[28] Nehru K., Jang Y., Oh S., Dallemer F., Nam W., Kim J. (2008). Oxidation of hydroquinones by a nonheme iron(iv)-oxo species. Inorg. Chim. Acta.

[29] Marteau C., Guitard R., Penverne C., Favier D., Nardello-Rataj V., Aubry J.M. (2016). Boosting effect of ortho-propenyl substituent on the antioxidant activity of natural phenols. Food Chem..

[30] Leopoldini M., Marino T., Russo N., Toscano M. (2004). Antioxidant properties of phenolic compounds: H-atom versus electron transfer mechanism. J. Phys. Chem. A.

[31] Nenadis N., Tsimidou M.Z. (2012). Contribution of dft computed molecular descriptors in the study of radical scavenging activity trend of natural hydroxybenzaldehydes and corresponding acids. Food Res. Int..

[32] Chen Y., Xiao H., Zheng J., Liang G. (2015). Structure-thermodynamics-antioxidant activity relationships of selected natural phenolic acids and derivatives: An experimental and theoretical evaluation. PLoS ONE.

[33] Amorati R., Pedulli G.F., Cabrini L., Zambonin L., Landi L. (2006). Solvent and ph effects on the antioxidant activity of caffeic and other phenolic acids. J. Agric. Food Chem..

[34] Foti M.C., Daquino C., Mackie I.D., DiLabio G.A., Ingold K.U. (2008). Reaction of phenols with the 2,2-diphenyl-1-picrylhydrazyl radical: Kinetics and dft calculations applied to determine aro-h bond dissociation enthalpies and reaction mechanism. J. Org. Chem..

[35] Pino E., Campos A.M., Lopez-Alarcon C., Aspee A., Lissi E. (2006). Free radical scavenging capacity of hydroxycinnamic acids and related compounds. J. Phys. Org. Chem..

[36] Amic D., Lucic B. (2010). Reliability of bond dissociation enthalpy calculated by the pm6 method and experimental teac values in antiradical qsar of flavonoids. Bioorg. Med. Chem..

[37] Zhang H.-Y., Wang L.-F. (2004). Theoretical elucidation of structure-activity relationship for coumarins to scavenge peroxyl radical. J. Mol. Struct.Theochem.

[38] Saito M., Sakagami H., Fujisawa S. (2003). Cytotoxicity and apoptosis induction by butylated hydroxyanisole (bha) and butylated hydroxytoluene (bht). Anticancer Res..

[39] Choe E., Min D.B. (2009). Mechanisms of antioxidants in the oxidation of foods. Compr. Rev. Food Sci. Food Saf..

[40] Natella F., Nardini M., Di Felice M., Scaccini C. (1999). Benzoic and cinnamic acid derivatives as antioxidants:  Structure−activity relation. J. Agric. Food Chem..

[41] Musialik M., Kuzmicz R., Pawlowski T.S., Litwinienko G. (2009). Acidity of hydroxyl groups: An overlooked influence on antiradical properties of flavonoids. J. Org. Chem..

[42] Hudson B.J.F., Lewis J.I. (1983). Polyhydroxy flavonoid antioxidants for edible oils: Structural criteria for activity. Food Chem..

[43] Bors W., Heller W., Michel C., Saran M. (1990). Flavonoids as antioxidants: Determination of radical-scavenging efficiencies. Methods Enzymol..

[44] Garcia Aranzazu M., Ruiz-Mendez V., Romero C., Brenes M. (2006). Effect of refining on the phenolic composition of crude olive oils. J. Am. Oil Chem. Soc..

[45] Hsu D.-Z., Chu P.-Y., Chandrasekaran V.R.M., Liu M.-Y. (2009). Sesame lignan sesamol protects against aspirin-induced gastric mucosal damage in rats. J. Funct. Foods.

[46] Erkan N., Ayranci G., Ayranci E. (2008). Antioxidant activities of rosemary (*Rosmarinus officinalis* L.) extract, blackseed (*Nigella sativa* L.) essential oil, carnosic acid, rosmarinic acid and sesamol. Food Chem..

[47] Guitard R., Paul J.F., Nardello V., Aubry J.M. (2016). Myricetin, rosmarinic and carnosic acid as superior natural antioxidant alternatives to a-tocopherol for the preservation of omega-3 oils. Food Chem..

[48] Beghdad M.C., Benammar C., Bensalah F., Sabri F.-Z., Belarbi M., Chemat F. (2014). Antioxidant activity, phenolic and flavonoid content in leaves, flowers, stems and seeds of mallow (*Malva sylvestris* L.) from north western of algeria. Afr. J. Biotechnol..

[49] Brand-Williams W., Cuvelier M.E., Berset C. (1995). Use of a free radical method to evaluate antioxidant activity. Food Sci. Technol..

[50] Antolovich M., Prenzler P.D., Patsalides E., McDonald S., Robards K. (2002). Methods for testing antioxidant activity. Analyst.

[51] Huang D., Ou B., Prior R.L. (2005). The chemistry behind antioxidant capacity assays. J. Agric. Food Chem..

[52] Mishra K., Ojha H., Chaudhury N.K. (2012). Estimation of antiradical properties of antioxidants using DPPH[rad] assay: A critical review and results. Food Chem..

[53] Goupy P., Bautista-Ortin A.-B., Fulcrand H., Dangles O. (2009). Antioxidant activity of wine pigments derived from anthocyanins: Hydrogen transfer reactions to the DPPH radical and inhibition of the heme-induced peroxidation of linoleic acid. J. Agric. Food Chem..

[54] Goupy P., Dufour C., Loonis M., Dangles O. (2003). Quantitative kinetic analysis of hydrogen transfer reactions from dietary polyphenols to the dpph radical. J. Agric. Food Chem..

[55] Encinar J.M., Pardal A., Martinez G. (2012). Transesterification of rapeseed oil in subcritical methanol conditions. Fuel Process. Technol..

[56] Lucarini M., Pedulli G.F. (2010). Free radical intermediates in the inhibition of the autoxidation reaction. Chem. Soc. Rev..

[57] Brigati G., Lucarini M., Mugnaini V., Pedulli G.F. (2002). Determination of the substituent effect on the o-h bond dissociation enthalpies of phenolic antioxidants by the epr radical equilibration technique. J. Org. Chem..

[58] Lucarini M., Pedrielli P., Pedulli G.F., Cabiddu S., Fattuoni C. (1996). Bond dissociation energies of O-H bonds in substituted phenols from equilibration studies. J. Org. Chem..

[59] Foti M.C., Johnson E.R., Vinqvist M.R., Wright J.S., Barclay L.R.C., Ingold K.U. (2002). Naphthalene diols: A new class of antioxidants intramolecular hydrogen bonding in catechols, naphthalene diols, and their aryloxyl radicals. J. Org. Chem..

[60] Li J., Bi Y., Liu W., Sun S., Liu C., Ma S. (2014). Effect of acid value on tbhq and bht losses in heating oils: Identification of the esterification products of tbhq and free fatty acids. J. Am. Oil Chem. Soc..

