# Supplementary material for: Theoretical and Kinetic Tools for Selecting Effective Antioxidants: Application to the Protection of Omega-3 Oils with Natural and Synthetic Phenols
Source: Int J Mol Sci. 2016 Jul 29;17(8):1220. doi: 10.3390/ijms17081220 (PMC5000618; doi:10.3390/ijms17081220)
Supplement: Supplementary file 1 [file ijms-17-01220-s001.pdf]

Int. J. Mol. Sci. **2016**, *17*, 1220; doi:10.3390/ijms17081220

Supplementary Materials: Theoretical and Kinetic Tools for Selecting Effective Antioxidants: Application to the Protection of Omega-3 Oils with Natural and Synthetic Phenols

Romain Guitard, Véronique Nardello-Rataj and Jean-Marie Aubry

Table S1. Bond Dissociation Enthalpies BDEs (O-H).

Number	Name	Structure	Site	BDE O-H (kcal·mol^{-1})
		**Synthetic antioxidants**		
**1**	5-*Tert*-butyl-pyrogallol	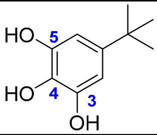	**4**	**66.6**
			3,5	74.1
**2**	Pyrogallol	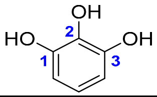	**2**	**68.0**
			1,3	74.4
**3**	Hydroxyquinol	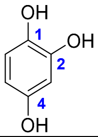	**1**	**69.1**
			2	72.1
			4	75.9
**4**	Propyl gallate	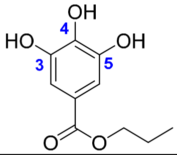	**4**	**69.6**
			3,5	75.7
**5**	BHA	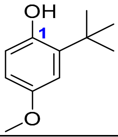	**1**	**72.3**
**6**	4-*Tert*-butyl-catechol	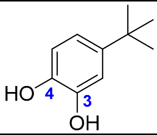	**4**	**72.3**
			3	73.2
**7**	BHT	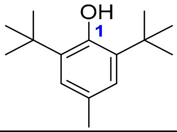	**1**	**72.4**
**8**	TBHQ	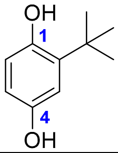	**1**	**74.3**
			4	76.7
**9**	*o*-*Tert*-butyl-*p*-cresol	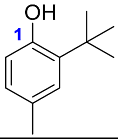	**1**	**77.4**
**10**	Phloroglucinol	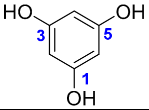	**1,3,5**	**83.0**

Table S1. *Cont.*

Number	Name	Structure	Site	BDE O-H (kcal·mol⁻¹)
		**Tocopherols**		
**11**	α-Tocopherol	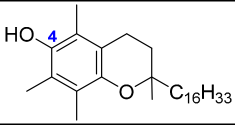	**4**	**69.1**
**12**	β-Tocopherol	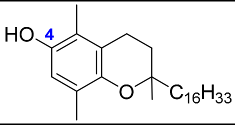	**4**	**73.4**
**13**	γ-Tocopherol	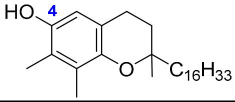	**4**	**73.5**
**14**	δ-Tocopherol	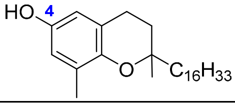	**4**	**75.4**
		**Derivatives of hydroxybenzoic acids**		
**15**	Gallic acid	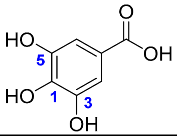	**4**	**70.2**
			3,5	76.3
**16**	Protocatechuic acid	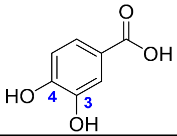	**4**	**75.5**
			3	75.6
**17**	Syringic acid	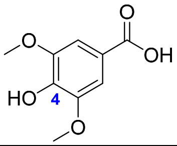	**4**	**78.1**
**18**	Ellagic acid	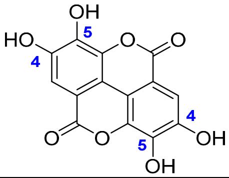	**4**	**78.4**
			5	78.9
**19**	Gentisic acid	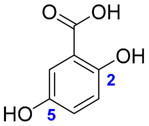	**5**	**79.5**
			2	84.6
**20**	Vanillic acid	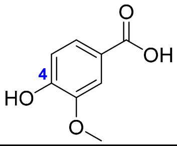	**4**	**83.1**
**21**	PHBA	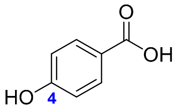	**4**	**84.7**
**22**	Salicylic acid	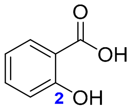	**1**	**95.2**

Table S1. *Cont.*

Number	Name	Structure	Site	BDE O-H (kcal·mol^{-1})
**Hydroxycinnamic acid derivatives**				
**23**	Rosmarinic acid	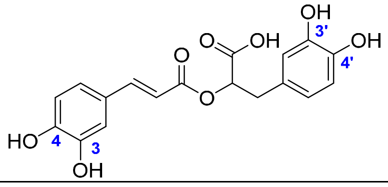	**4**	**69.2**
			3	75.2
			4′	72.4
			3′	75.9
**24**	Caffeic acid	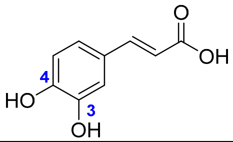	**4**	**72.1**
			3	74.6
**25**	Chlorogenic acid	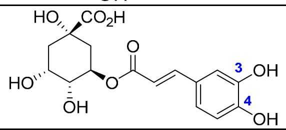	**4**	**73.4**
			3	75.9
**26**	Sinapic acid	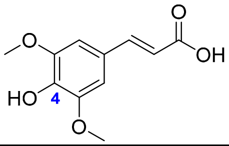	**4**	**75.4**
**27**	Ferulic acid	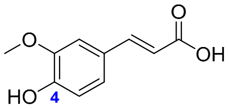	**4**	**79.7**
**28**	*o*-Coumaric acid	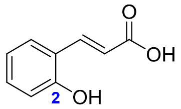	**2**	**80.1**
**29**	*p*-Coumaric acid	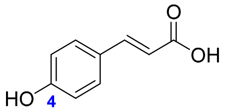	**4**	**80.5**
**30**	*m*-Coumaric acid	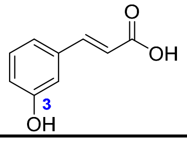	**3**	**84.4**

Table S1. *Cont.*

Number	Name	Structure	Site	BDE O-H (kcal·mol⁻¹)
**Flavonols**				
**31**	Gossypetin	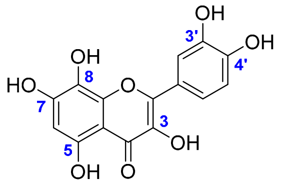	3′	75.1
			4′	72.4
			3	79.8
			5	88.2
			7	71.7
			**8**	**66.6**
**32**	Myricetin	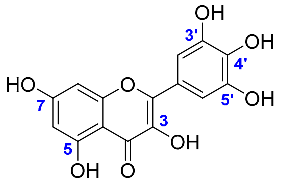	3′	76.1
			**4′**	**67.4**
			5′	76.3
			3	79.6
			5	93.8
			7	85.8
**33**	Azaleatin	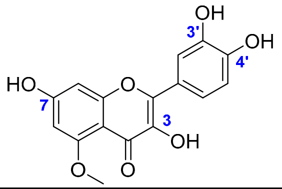	3′	73.9
			**4′**	**71.1**
			3	81.9
			7	84.6
**34**	Quercetin	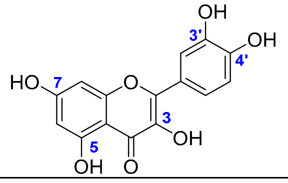	3′	74.3
			**4′**	**71.8**
			3	80.4
			5	95.0
			7	84.9
**35**	Fisetin	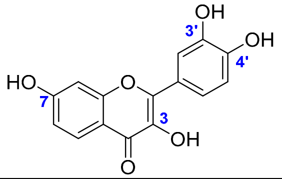	3′	75.3
			**4′**	**72.3**
			3	80.9
			7	83.9
**36**	Laricitrin	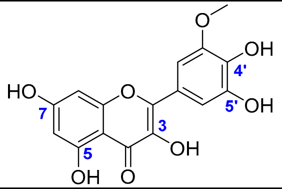	**4′**	**72.5**
			5′	79.4
			3	80.6
			5	94.9
			7	84.7
**37**	Syringetin	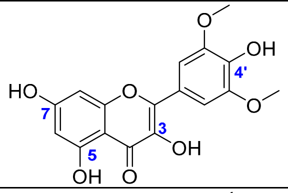	**4′**	**75.7**
			3	79.0
			5	93.7
			7	85.4
**38**	Rhamnazin	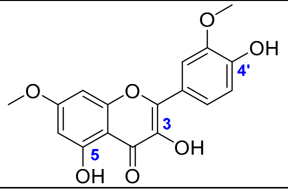	**4′**	**79.6**
			3	80.8
			5	93.3
**39**	Kaempferide	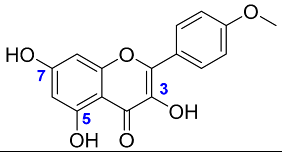	**3**	**79.8**
			5	93.9
			7	85.4

Table S1. *Cont.*

Number	Name	Structure	Site	BDE O-H (kcal·mol^{-1})
**40**	Isorhamnetin	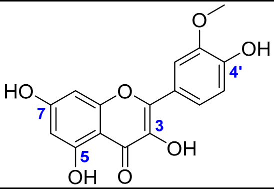	**4′**	**79.8**
			3	80.2
			5	94.0
			7	85.1
**41**	Morin	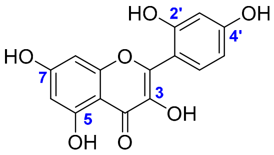	2′	82.9
			4′	82.2
			**3**	**79.8**
			5	94.6
			7	85.8
**42**	Kaempferol	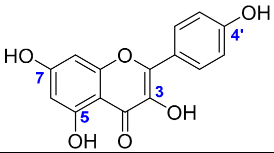	**4′**	**80.1**
			3	80.5
			5	94.8
			7	85.2
**43**	Galangin	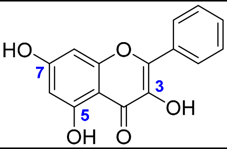	**3**	**81.2**
			5	94.0
			7	86.2
		**Flavones**		
**44**	Luteolin	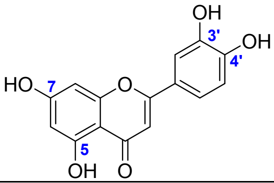	3′	74.9
			**4′**	**73.1**
			5	113.2
			7	86.6
**45**	Apigenin	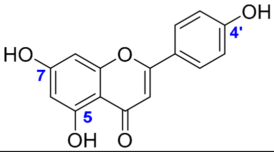	**4′**	**82.1**
			5	98.4
			7	86.5
		**Flavanonols**		
**46**	Taxifolin	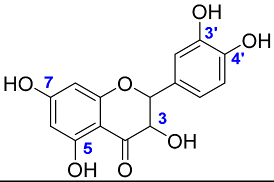	**4′**	**73.2**
			3′	73.5
			3	103.4
			5	94.8
			7	87.3
**47**	Aromadedrin	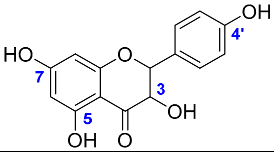	4′	**82.3**
			3	97.8
			5	95.7
			7	88.4

Table S1. *Cont.*

Number	Name	Structure	Site	BDE O-H (kcal·mol⁻¹)
**Flavanones**				
**48**	Eriodictyol	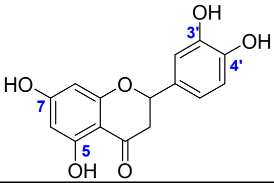	3′	73.8
			**4′**	**73.6**
			5	103.8
			7	87.6
**49**	Homoeriodictyol	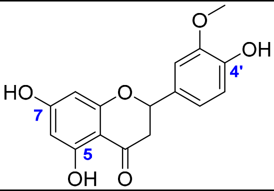	**4′**	**80.8**
			5	96.8
			7	87.8
**50**	Hesperetin	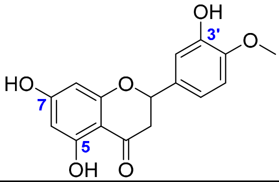	**3′**	**82.2**
			5	103.6
			7	87.7
**51**	Naringenin	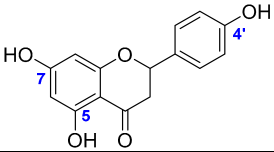	**4′**	**82.4**
			5	96.7
			7	87.8
**Isoflavones**				
**52**	Glycitein	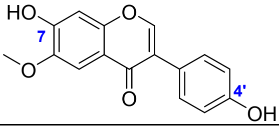	**4′**	**80.1**
			7	84.1
**53**	Genistein	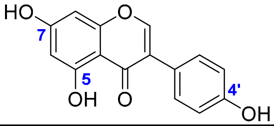	**4′**	**81.0**
			5	112.5
			7	87.3
**54**	Daidzein	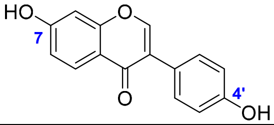	**4′**	**81.9**
			7	84.8

Table S1. *Cont.*

Number	Name	Structure	Site	BDE O-H (kcal·mol^{-1})
		**Catechins**		
**55**	Epigallocatechin gallate	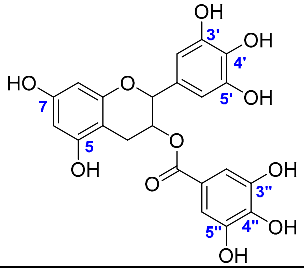	3′	73.5
			**4′**	**66.5**
			5′	73.1
			3′′	75.8
			4′′	68.6
			5′′	75.4
			5	79.6
			7	83.4
**56**	Gallocatechin	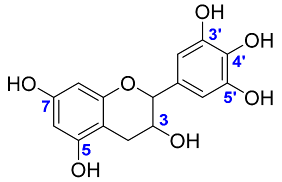	3′	75.8
			**4′**	**68.6**
			5′	75.2
			3	100.8
			5	79.2
			7	82.0
**57**	Catechin	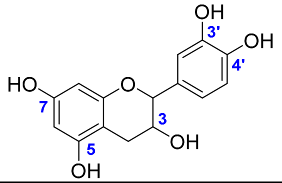	3′	82.5
			**4′**	**74.4**
			3	102.4
			5	79.1
			7	82.9
		**Stilbenes**		
**58**	Piceatannol	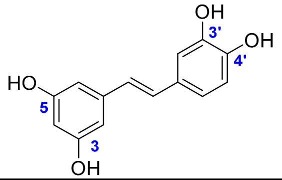	**4′**	**68.7**
			5′	71.2
			3	82.1
			5	82.9
**59**	Resveratrol	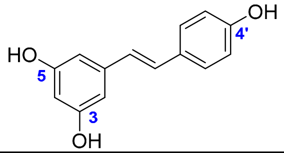	**4′**	**76.7**
			3	82.7
			5	82.0
		**Eugenol derivatives**		
**60**	Isoeugenol	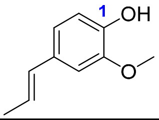	**1**	**76.6**
**61**	Eugenol	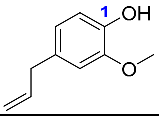	**1**	**80.2**
		**Antioxidants found in olive oil**		
**62**	Hydroxytyrosol	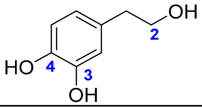	**4**	**72.1**
			3	72.8
			2	97.4
**63**	Catechol	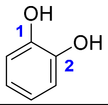	**1,2**	**73.4**
**64**	Tyrosol	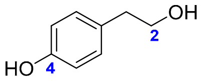	**4**	**81.0**
			2	97.5

Table S1. *Cont.*

Number	Name	Structure	Site	BDE O-H (kcal·mol⁻¹)
**Lignans**				
**65**	Sesamol	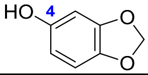	**4**	**75.1**
**Coumarins**				
**66**	Methylesculetin	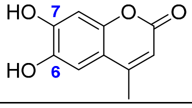	**6**	**72.0**
			7	73.4
**67**	Aesculetin	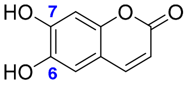	**6**	**72.5**
			7	73.0
**68**	Nordalbergin	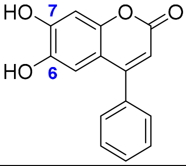	**6**	**72.6**
			7	73.2
**Antioxidants found in Rosemary extract**				
**69**	Carnosol	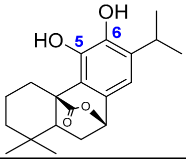	**6**	**70.7**
			5	71.6
**70**	Carnosic acid	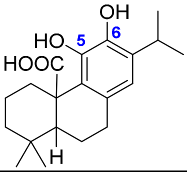	**6**	**70.8**
			5	71.4

Table S2. Kinetic rate constants of hydrogen transfer from phenolic antioxidants to the $DPPH^\bullet$ radical in toluene at 20 °C.

Phenols	Methods	$10^4 \times [DPPH^\bullet]$ (M)	$10^3 \times [ArOH]_0$ (M)	$10^3 \times k_{app}$	k ($M^{-1} \cdot s^{-1}$)
1	SOK	1.25	0.125	809.2<br>802.5<br>805.6	9480
4	SOK	1.25	0.125	107.7<br>104.9<br>103.9	1240
5	SOK	1.25	0.125	14.6<br>15.8<br>16.8	184
6	SOK	1.25	0.125	67.4<br>64.5<br>65.9	776
7	FOK	1.25	2.0<br>3.0<br>4.0<br>5.0	0.63<br>0.76<br>0.98<br>1.16	0.18
8	SOK	1.25	0.125	51.1<br>51.3<br>51.4	600

Table S2. *Cont.*

Phenols	Methods	$10^4 \times [DPPH^\bullet]$ (M)	$10^3 \times [ArOH]_0$ (M)	$10^3 \times k_{app}$	k ($M^{-1} \cdot s^{-1}$)
9	FOK	1.25	0.9<br>1.35<br>1.80<br>2.25	0.38<br>0.58<br>0.70<br>0.88	0.36
11	SOK	1.25	0.125	231.4<br>226.9<br>227.6	2690
17	SOK	1.25	0.125	0.90<br>0.92<br>0.87	10.6
20	SOK	1.25	0.125	0.12<br>0.12<br>0.12	1.4
20	FOK	1.25	2.0<br>3.0<br>4.0<br>5.0	2.03<br>3.04<br>3.92<br>5.01	1.0
26	SOK	1.25	0.125	12.2<br>15.9<br>14.1	165
27	SOK	1.25	0.125	0.65<br>0.76<br>0.73	8.4
60	SOK	1.25	0.125	3.01<br>3.29<br>3.33	38
61	FOK	1.25	0.9<br>1.35<br>1.8<br>2.25	2.58<br>3.88<br>5.23<br>6.23	2.7
61	SOK	1.25	0.125	0.34<br>0.32<br>0.33	3.9
62	SOK	1.25	0.125	88.6<br>90.9<br>92.9	1070
63	SOK	1.25	0.125	34.2<br>34.3<br>34.8	400
65	SOK	1.25	0.125	21.4<br>21.4<br>21.6	250
69	SOK	1.25	0.125	132.8<br>145.6<br>149.8	1680
70	SOK	1.25	0.125	54.5<br>54.3<br>54.0	640

Table S3. Determination of the stoichiometric numbers (σ_{exp}) of the hydrogen transfer from phenols to the DPPH• radical in toluene at 20 °C at 515 nm, [DPPH•] = 1.5×10^{-4} mol·L−1.

Phenols	$10^5 \times [ArOH]_0$ (M)	A_0	A_f	σ_{exp}
**1**	2.07	1.60	1.09	2.1
**4**	2.07	1.60	0.64	3.9
**5**	2.07	1.60	1.12	2.0
**6**	2.07	1.60	0.98	2.5
**7**	2.07	1.60	1.10	2.0
**8**	2.07	1.60	1.12	2.0
**9**	2.07	1.60	0.98	2.5
**11**	2.07	1.60	1.12	2.0
**15 ***	2.07	1.60	0.46	5.0
**16 ***	2.07	1.60	1.18	1.8
**17**	2.07	1.60	1.33	1.1
**23 ***	2.07	1.60	0.65	4.1
**24 ***	2.07	1.63	1.17	2.0
**25 ***	2.07	1.60	1.17	1.9
**26**	2.07	1.60	1.10	1.4
**27**	2.07	1.60	1.15	1.8
**32 ***	2.07	1.61	0.83	3.4
**34 ***	2.07	1.60	1.17	1.9
**55 ***	2.07	1.60	0.35	5.4
**58 ***	2.07	1.60	1.12	2.0
**59 ***	2.07	1.60	1.40	0.9
**60**	2.07	1.60	1.38	0.9
**61**	2.07	1.60	1.08	2.1
**62**	2.07	1.60	1.12	2.0
**63**	2.07	1.60	1.13	1.9
**65**	2.07	1.60	1.10	2.1
**67 ***	2.07	1.61	1.11	2.1
**69**	2.07	1.60	1.14	1.9
**70**	2.07	1.60	1.12	2.0

*: ethyl acetate used as solvent; nd: not determined

Table S4. Induction periods (IP) and oxidation rates (R_{ox}) for the inhibition of FAMEs linseed oil oxidation by phenolic antioxidants during the Rapidoxy® test.

Phenols	Induction Period IP (min)	IP Average (min)	Std. dev. IP (min)	Oxidation rate R_{ox} (mM·min−1)	R_{ox} Average (mM·min−1)	Std. dev. R_{ox} (mM·min−1)
	230			0.10		
**1**	240	234	5	0.04	0.06	0.03
	232			0.04		
	170			0.25		
**4**	150	162	11	0.28	0.26	0.02
	167			0.25		
	169			0.35		
**5**	166	167	2	0.40	0.35	0.05
	165			0.31		
	220			0.40		
**6**	210	220	10	0.36	0.37	0.02
	230			0.36		
	132			0.45		
**7**	132	131	1	0.37	0.44	0.07
	130			0.51		

Table S4. *Cont.*

Phenols	Induction Period IP (min)	IP Average (min)	Std. dev. IP (min)	Oxidation rate R_{ox} (mM·min^{-1})	R_{ox} Average (mM·min^{-1})	Std. dev. R_{ox} (mM·min^{-1})
	43			0.47		
**8**	43	45	2	0.56	0.53	0.06
	45			0.57		
	56			0.76		
**9**	58	56	2	0.76	0.77	0.02
	54			0.79		
	177			0.17		
**11**	179	177	2	0.18	0.17	0.01
	176			0.16		
	178			0.29		
**15**	172	178	6	0.30	0.32	0.04
	184			0.37		
	50			0.59		
**16**	46	50	4	0.62	0.62	0.03
	53			0.65		
	42			0.79		
**17**	38	37	6	0.86	0.76	0.11
	31			0.64		
	5			1.00		
**20**	5	5	1	1.08	1.03	0.04
	6			1.02		
	7			1.16		
**21**	6	6	1	1.18	1.20	0.05
	5			1.26		
	250			0.26		
**23**	260	262	14	0.28	0.27	0.01
	277			0.27		
	150			0.34		
**24**	142	148	5	0.36	0.36	0.02
	152			0.37		
	139			0.47		
**25**	136	138	2	0.48	0.48	0.02
	138			0.50		
	56			0.62		
**26**	58	54	6	0.52	0.57	0.05
	47			0.56		
	26			0.80		
**26**	28	28	2	0.82	0.82	0.02
	30			0.84		
	269			0.13		
**32**	252	262	9	0.10	0.11	0.02
	264			0.11		
	131			0.33		
**34**	130	135	8	0.35	0.34	0.01
	144			0.35		
	438			0.04		
**55**	477	476	37	0.09	0.08	0.03
	512			0.10		
	313			0.28		
**58**	321	313	8	0.30	0.29	0.04
	306			0.28		
	64			0.72		
**59**	68	67	2	0.69	0.68	0.04
	68			0.64		
	48			0.72		
**60**	48	49	1	0.74	0.72	0.02
	50			0.71		
	25			0.90		
**61**	28	27	2	0.99	0.93	0.05
	27			0.91		

Table S4. *Cont.*

Phenols	Induction Period IP (min)	IP Average (min)	Std. dev. IP (min)	Oxidation rate R_{ox} (mM·min^{-1})	R_{ox} Average (mM·min^{-1})	Std. dev. R_{ox} (mM·min^{-1})
	169			0.29		
**62**	175	172	3	0.32	0.30	0.02
	173			0.30		
	145			0.46		
**63**	150	147	3	0.46	0.46	0.01
	146			0.47		
	164			0.57		
**65**	160	161	2	0.55	0.55	0.01
	160			0.55		
	120			0.50		
**67**	111	112	8	0.53	0.50	0.03
	104			0.48		
	162			0.35		
**69**	163	166	6	0.34	0.35	0.01
	173			0.36		
	229			0.32		
**70**	222	230	8	0.28	0.29	0.02
	238			0.28		

2 mL of FAMEs linseed oil, [phenols]$_0$ = 0.5 mM, T = 90°C, P = 450 kPa, Std. dev. = Standard deviation.

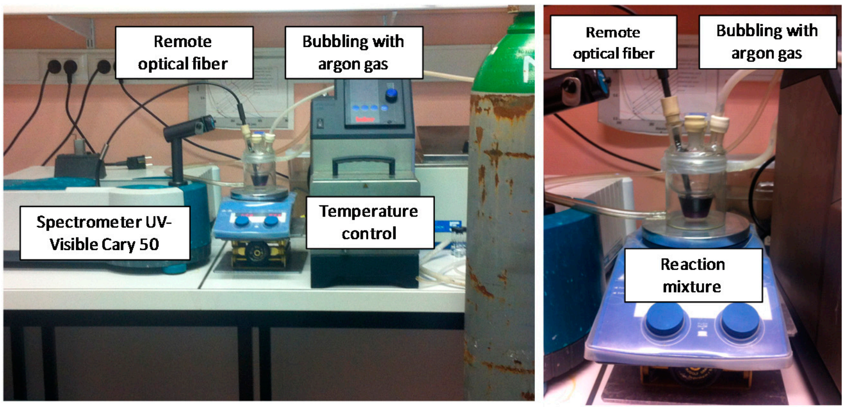

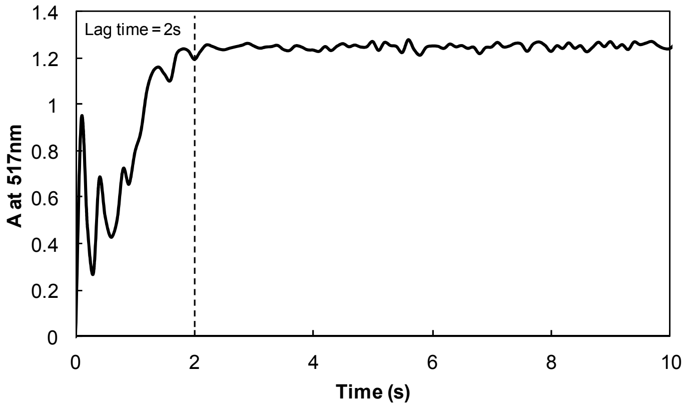

Figure S1. Equipment for UV-visible analysis and evolution of the absorbance of DPPH$^\bullet$ radical at 515 nm (0.12 mM) without phenol in toluene at 20 °C (visualization of the lag time).

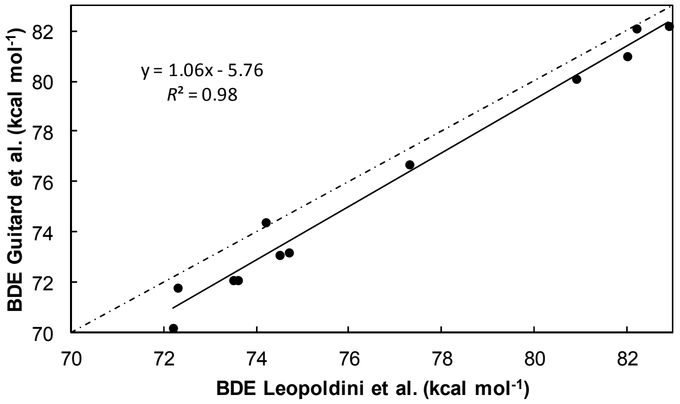

Figure S2. Correlation of BDEs obtained by Guitard et al. and Leopoldini et al. [22].

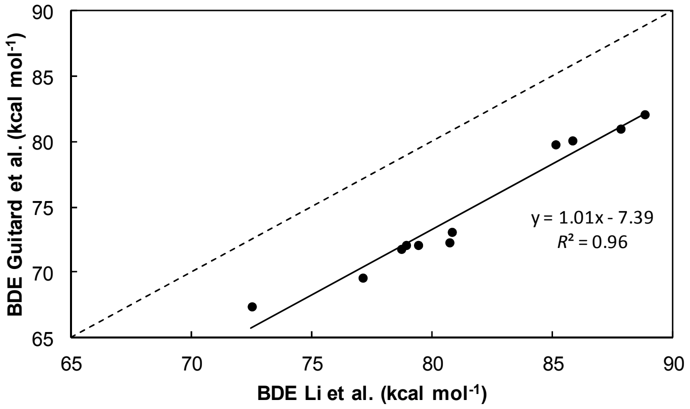

Figure S3. Correlation of BDEs obtained by Guitard et al. and Li et al. [23].

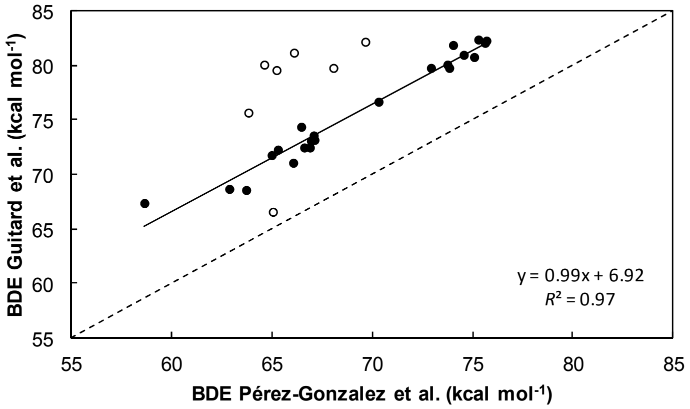

Figure S4. Correlation of BDEs obtained by Guitard et al. and Pérez-Gonzalez et al. [20].
